# Supplementary material for: Optogenetic engineering to probe the molecular choreography of STIM1-mediated cell signaling
Source: Nat Commun. 2020 Feb 25;11:1039. doi: 10.1038/s41467-020-14841-9 (PMC7042325; doi:10.1038/s41467-020-14841-9)
Supplement: Supplementary file 1 — Supplementary Information [file 41467_2020_14841_MOESM1_ESM.pdf]

Supporting information for

Optogenetic engineering to probe the molecular choreography of STIM1-mediated cell signaling

Guolin Ma, Lian He, Shuzhong Liu, Jiansheng Xie, Zixian Huang, Ji Jing, Yi-Tsang Lee, Rui Wang, Hesheng Luo, Weidong Han, Yun Huang, Yubin Zhou

Contents:

Supplementary Figures 1-19

Supplementary Table 1

Supplementary References

a

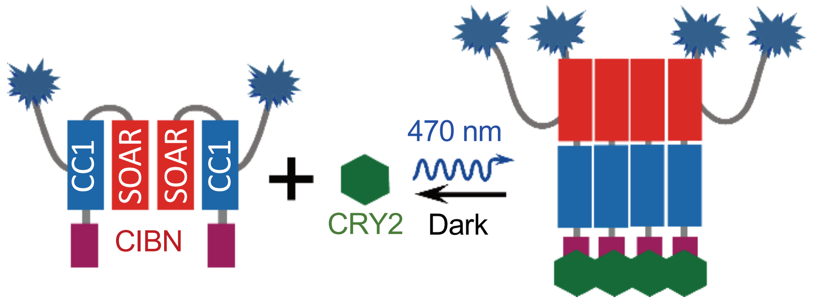

b

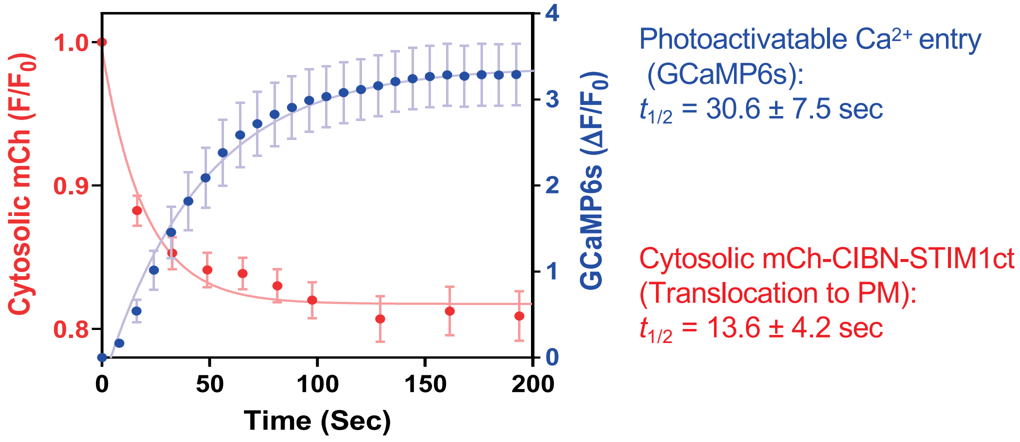

Supplementary Figure 1 | Use of CRY2 as an optical crosslinker to trigger the cytosol-to-PM translocation of CIBN-STIM1ct and the subsequent Ca^{2+} entry through ORAI channels. (Related to **Fig. 1**)

(**a**) Schematic of the design. (**b**) Time courses showing the cytosol-to-PM translocation of CIBN-STIM1ct (red) and light-elicited Ca^{2+} influx (blue) in HeLa cells co-transfected with CFP-ORAI1, CRY2-P2A-mCh-CIBN-STIM1ct and GCaMP6s. The corresponding half-lives ($t_{1/2}$) were listed on the right. n = 30 cells from three independent experiments (mean ± sem).

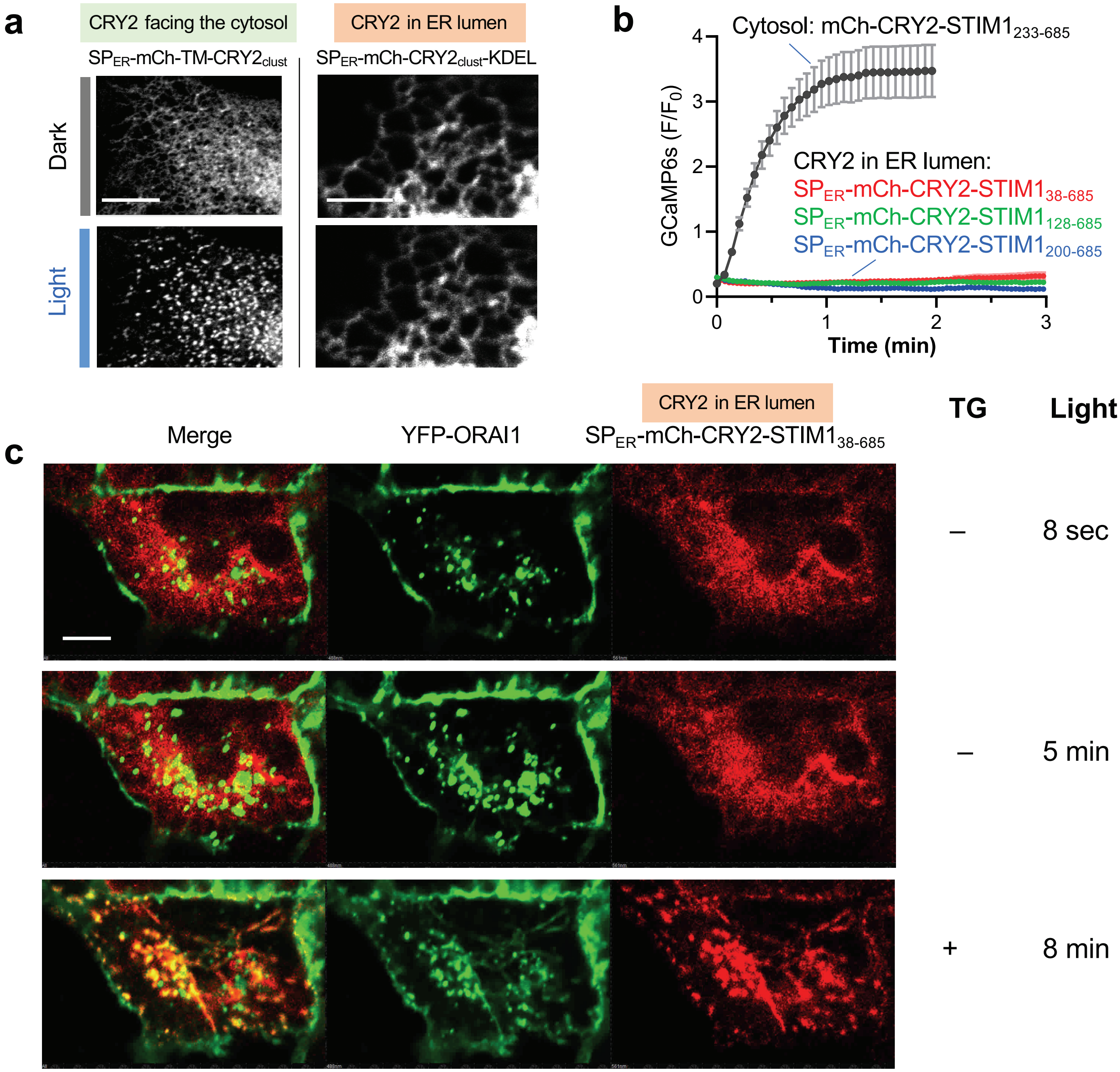

Supplementary Figure 2 | Photoactivation of CRY2 is environmentally sensitive. (Related to **Fig. 1**)

(**a**) Representative confocal images showing COS-7 cells expressing ER-anchored SP_{ER}-mCh-STIM1$_{209\text{-}214,\ TM}$-CRY2$_{clust}$ (***left***) or ER lumen-resident SP_{ER}-mCh-CRY2$_{clust}$-KDEL (***right***) before and after blue light illumination. Scale bar, 5 µm.

(**b**) Light-triggered Ca^{2+} responses for the indicated mCh-CRY2-STIM1ct variants. n = 30 cells. Data were shown as mean ± sem.

(**c**) Confocal images showing a HeLa cell cotransfected with YFP-ORAI1 and SP_{ER}-mCh-CRY2-STIM1$_{38\text{-}685}$ before and after light illumination (470 nm; 5 min) with and without TG stimulation. Scale bar, 5 µm.

NOTE: SP_{ER}-mCh-TM-CRY2$_{clust}$ (with CRY2 facing the cytosol), but not SP_{ER}-mCh-CRY2$_{clust}$-KDEL in the ER lumen, formed clusters along ER tubules after photo-stimulation. We reason that blue light illumination cannot effectively reduce CRY2 in the more oxidative ER lumen to photoactivate CRY2 (redox potential: -118 mV in the ER lumen[1] versus -300 mV in the cytosol[2-3]). Therefore, CRY2-STIM1 chimeras with CRY2 in the ER lumen invariably failed to photo-trigger Ca^{2+} influx.

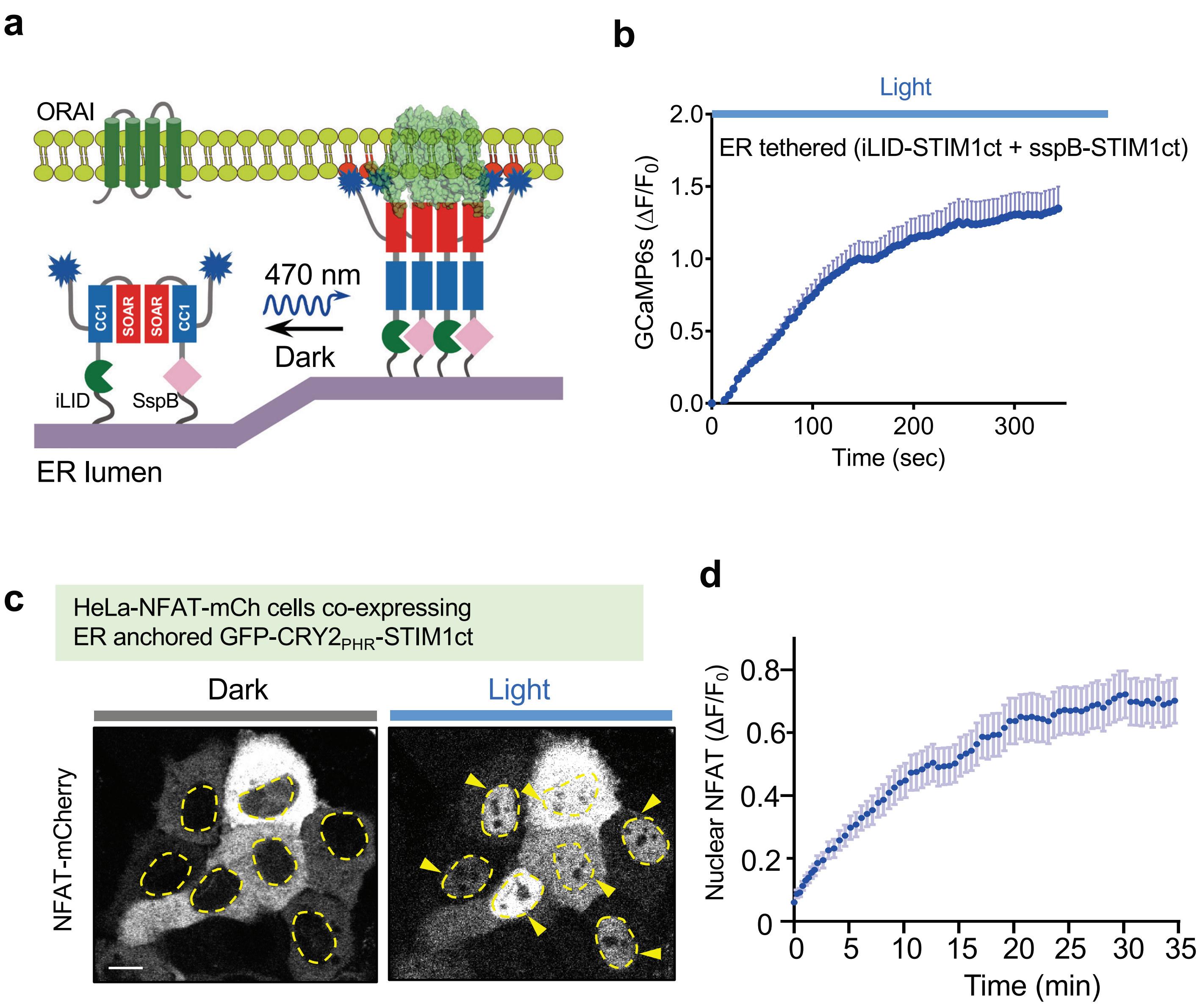

Supplementary Figure 3 | Optical control of homo- or heterodimerization of ER-anchored, engineered STIM1ct to trigger Ca^{2+} influx and NFAT nuclear entry. (Related to **Fig. 1**)

(**a-b**) Use of ER-tethered optical dimerizer (iLID + sspB) to drive STIM1ct activation with light.

(**a**) Schematic of the design. (**b**) A representative light-induced Ca^{2+} response curve in HeLa cells expressing iLID-STIM1ct and sspB-STIM1ct. n = 24 (mean ± sem).

(**c**) Light-induced nuclear translocation of NFAT-mCherry in HeLa cells expressing ER localized GFP-CRY2$_{PHR}$-STIM1ct. The HeLa cells were subjected to blue light illumination for 20 min (pulsed cycles of 30 s ON + 120 s OFF). Representative images were shown before and after light illumination (470 nm, 4 mW/cm^2, blue bar). Arrows: nuclear NFAT-mCherry (mCh). Scale bar, 10 µm.

(**d**) The time course of light-induced NFAT nuclear translocation shown in panel c. n = 12 cells (mean ± sem.).

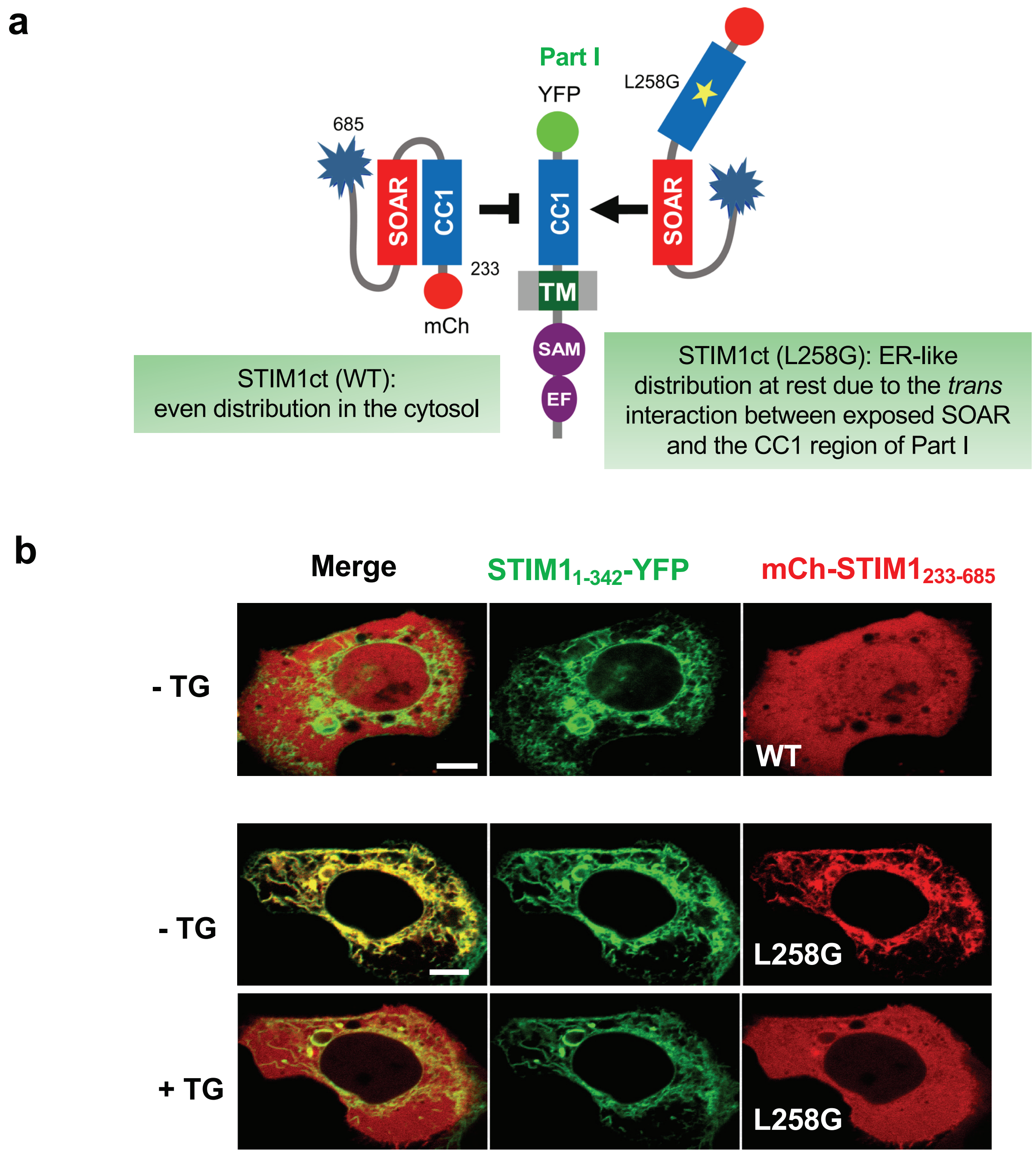

Supplementary Figure 4 | CC1-SOAR interaction mediates STIM1ct autoinhibition. (Related to **Fig. 2**)

(**a**) Schematic illustration of STIM1ct autoinhibition mediated by the interaction between CC1 and SOAR domains. ER anchored CC1 domain (Part I: STIM1$_{1\text{-}342}$-YFP) failed to capture mCh-tagged STIM1ct (WT) but tightly interacted with the STIM1ct-L258G mutant. The L258G mutation disrupts the intramolecular CC1-SOAR interaction (*cis*), thus exposing the SOAR domain to enable its *trans* interaction with CC1 in Part I.

(**b**) Representative confocal images of HeLa cells expressing ER anchored STIM1$_{1\text{-}342}$-YFP (Part I) and mCh-STIM1ct (WT, top panel) or STIM1ct-L258G (middle and bottom panel). 1 μM thapsigargin (TG) was used to trigger Ca^{2+} store depletion in the ER. Scale bar, 5 μm.

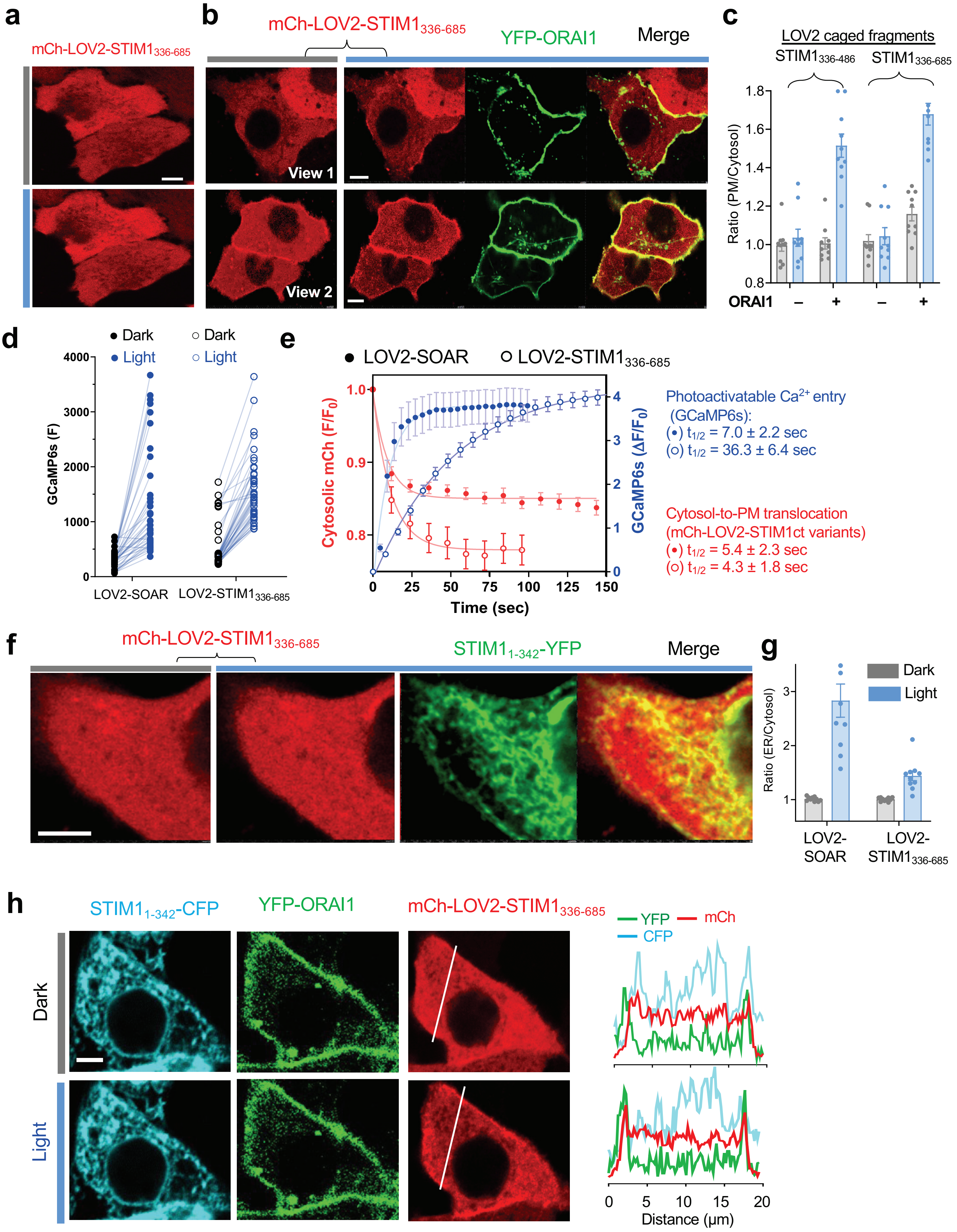
a
mCh-LOV2-STIM1336-685
b
mCh-LOV2-STIM1336-685
YFP-ORAI1
Merge
View 1
View 2
c
LOV2 caged fragments
STIM1336-486
STIM1336-685
Ratio (PM/Cytosol)
ORAI1
d
Dark
Light
GCaMP6s (F)
LOV2-SOAR
LOV2-STIM1336-685
e
LOV2-SOAR
LOV2-STIM1336-685
Cytosolic mCh (F/F0)
GCaMP6s (ΔF/F0)
Time (sec)
Photoactivatable Ca2+ entry
(GCaMP6s):
(•) t1/2 = 7.0 ± 2.2 sec
(○) t1/2 = 36.3 ± 6.4 sec
Cytosol-to-PM translocation
(mCh-LOV2-STIM1ct variants)
(•) t1/2 = 5.4 ± 2.3 sec
(○) t1/2 = 4.3 ± 1.8 sec
f
mCh-LOV2-STIM1336-685
STIM11-342-YFP
Merge
g
Dark
Light
Ratio (ER/Cytosol)
LOV2-
SOAR
LOV2-
STIM1336-685
h
STIM11-342-CFP
YFP-ORAI1
mCh-LOV2-STIM1336-685
Dark
Light
YFP
mCh
CFP
Distance (μm)

Supplementary Figure 5 | Characterization of LOV2 caged STIM1 fragments. (Related to **Fig. 2**)
Scale bar, 5 µm. Error bar denote sem.

(**a**) Representative confocal images of HeLa cells expressing mCh-LOV2-STIM1$_{336\text{-}685}$ before and after blue light illumination (470 nm, 5 min).

(**b**) Representative confocal images showing HeLa cells co-expressing mCh-LOV2-STIM1$_{336\text{-}685}$ and YFP-ORAI1 in the dark (two views with the bottom panel showing partial PM decoration), or after blue light stimulation (blue bar).

(**c**) Summary of the degrees of cytosol-to-PM translocation of the indicated constructs (LOV2-STIM1$_{336\text{-}486}$ [abbreviated as LOV2-SOAR] and LOV2-STIM1$_{336\text{-}685}$) in the absence or presence of YFP-ORAI expression. n = 10 cells.

(**d**) Comparison of cytosolic Ca^{2+} levels reported by GCaMP6s in HeLa cells expressing mCh-LOV2-SOAR (●) or mCh-LOV2-STIM1$_{336\text{-}685}$ (○) before and after blue light illumination. Some cells transfected with mCh-LOV2-STIM1$_{336\text{-}685}$ showed pre-activation with higher basal GCaMP6s signals in the dark. n = 38 cells.

(**e**) The time courses showing the kinetics of cytosol-to-PM translocation (red) and Ca^{2+} influx (blue) for mCh-LOV2-SOAR (●) or mCh-LOV2-STIM1$_{336\text{-}685}$ (○). HeLa cells were cotransfected with ORAI1-CFP and GCaMP6s. The half-lives ($t_{1/2}$) were listed on the right. n = 12 cells for PM translocation; n = 24 cells for the Ca^{2+} influx assay.

(**f**-**g**) Representative confocal images (**f**) of HeLa cells co-expressing mCh-LOV2-STIM1$_{336\text{-}685}$ and STIM1$_{1\text{-}342}$-YFP. The quantification of ER-bound versus cytosolic mCh-tagged protein was shown in panel **g**. n = 10 cells.

(**h**) Confocal images of HeLa cells co-expressing mCh-LOV2-STIM1$_{336\text{-}685}$ (red), YFP-ORAI1 (green) and STIM1$_{1\text{-}342}$-CFP (cyan). In the dark, mCh-LOV2-STIM1$_{336\text{-}685}$ was evenly distributed in the cytosol. Upon photostimulation, mCh-LOV2-STIM1$_{336\text{-}685}$ primarily translocated toward PM. The fluorescence intensities (YFP, green; mCh, red; CFP, cyan) across the dashed line were plotted to evaluate the degree of colocalization.

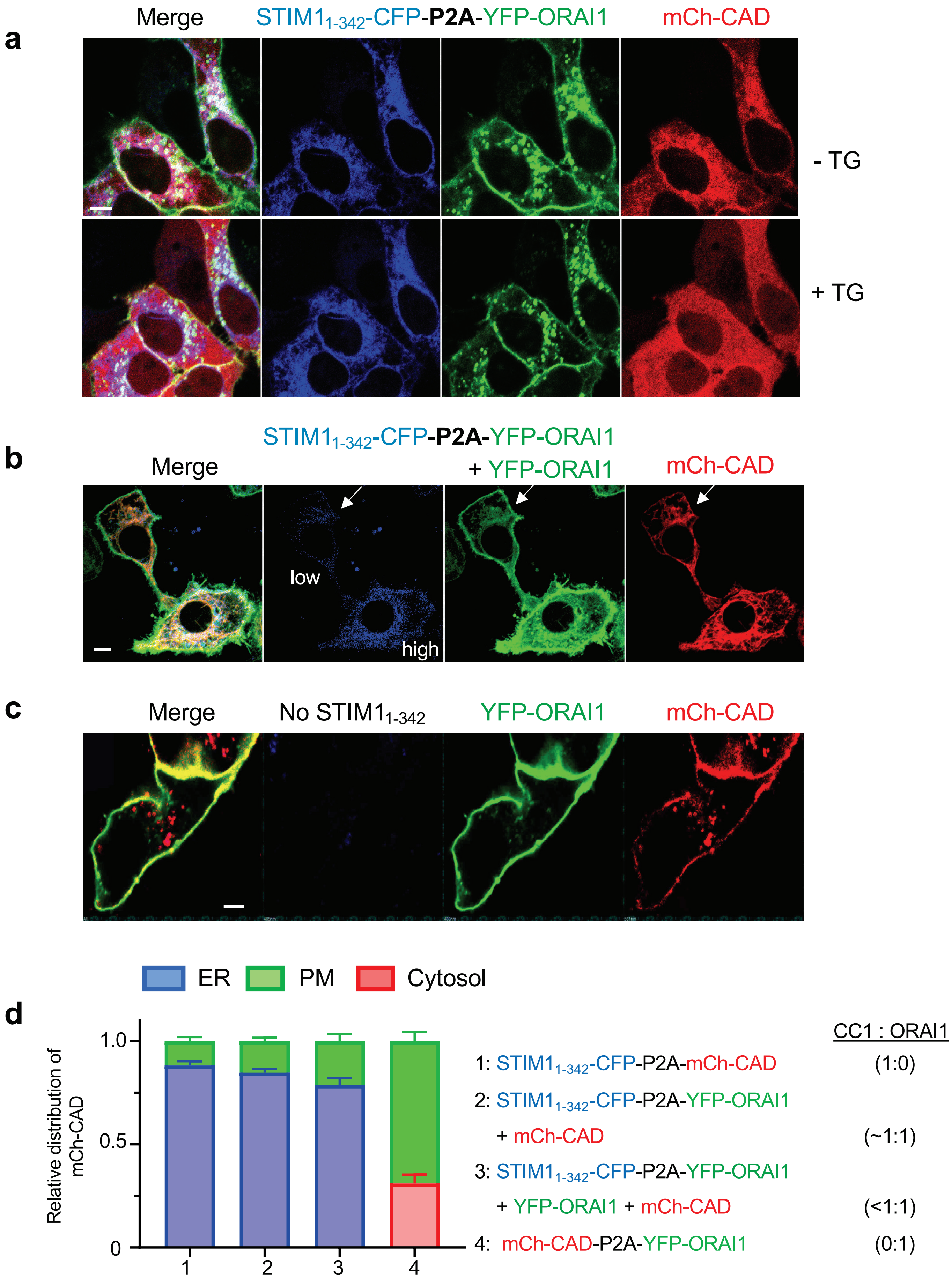
a
Merge
STIM1$_{1-342}$-CFP-P2A-YFP-ORAI1
mCh-CAD
- TG
+ TG
b
STIM1$_{1-342}$-CFP-P2A-YFP-ORAI1
Merge
+ YFP-ORAI1
mCh-CAD
low
high
c
Merge
No STIM1$_{1-342}$
YFP-ORAI1
mCh-CAD
d
ER
PM
Cytosol
Relative distribution of mCh-CAD
1.0
0.5
0
1
2
3
4
CC1 : ORAI1
1: STIM1$_{1-342}$-CFP-P2A-mCh-CAD (1:0)
2: STIM1$_{1-342}$-CFP-P2A-YFP-ORAI1 + mCh-CAD (~1:1)
3: STIM1$_{1-342}$-CFP-P2A-YFP-ORAI1 + YFP-ORAI1 + mCh-CAD (<1:1)
4: mCh-CAD-P2A-YFP-ORAI1 (0:1)

Supplementary Figure 6 | A 'tug-of-war' between ER-resident CC1 ($STIM1_{1-342}$) and PM-embedded ORAI1 to engage SOAR/CAD expressed in the cytosol. (Related to **Fig. 2**) Scale bar, 5 μm. Error bars denote sem.

(**a**) Representative confocal images of HeLa cells co-expressing $STIM1_{1-342}$-CFP-P2A-YFP-ORAI1 and mCh-CAD before and after 1 μM TG. mCh-CAD showed an ER-like tubular distribution at rest but was dispersed into the cytosol after TG-induced store depletion.

(**b**) Confocal images showing HeLa cells expressing $STIM1_{1-342}$-CFP-P2A-YFP-ORAI1, YFP-ORAI1 and mCh-CAD. mCh-CAD decorated both ER tubular structures and PM. The upper left cell had low expression of $STIM1_{1-342}$-CFP whereas the bottom right cell had higher $STIM1_{1-342}$-CFP levels (hence more ER-docked mCh-CAD).

(**c**) Confocal images of HeLa cells expressing mCh-CAD-P2A-YFP-ORAI1. mCh-CAD showed a predominant decoration surrounding the PM.

(d) Quantification of the relative subcellular distribution of mCh-CAD in HeLa cells expressing $STIM1_{1-342}$ and YFP-ORAI1 at varying ratios. n = 10 cells.

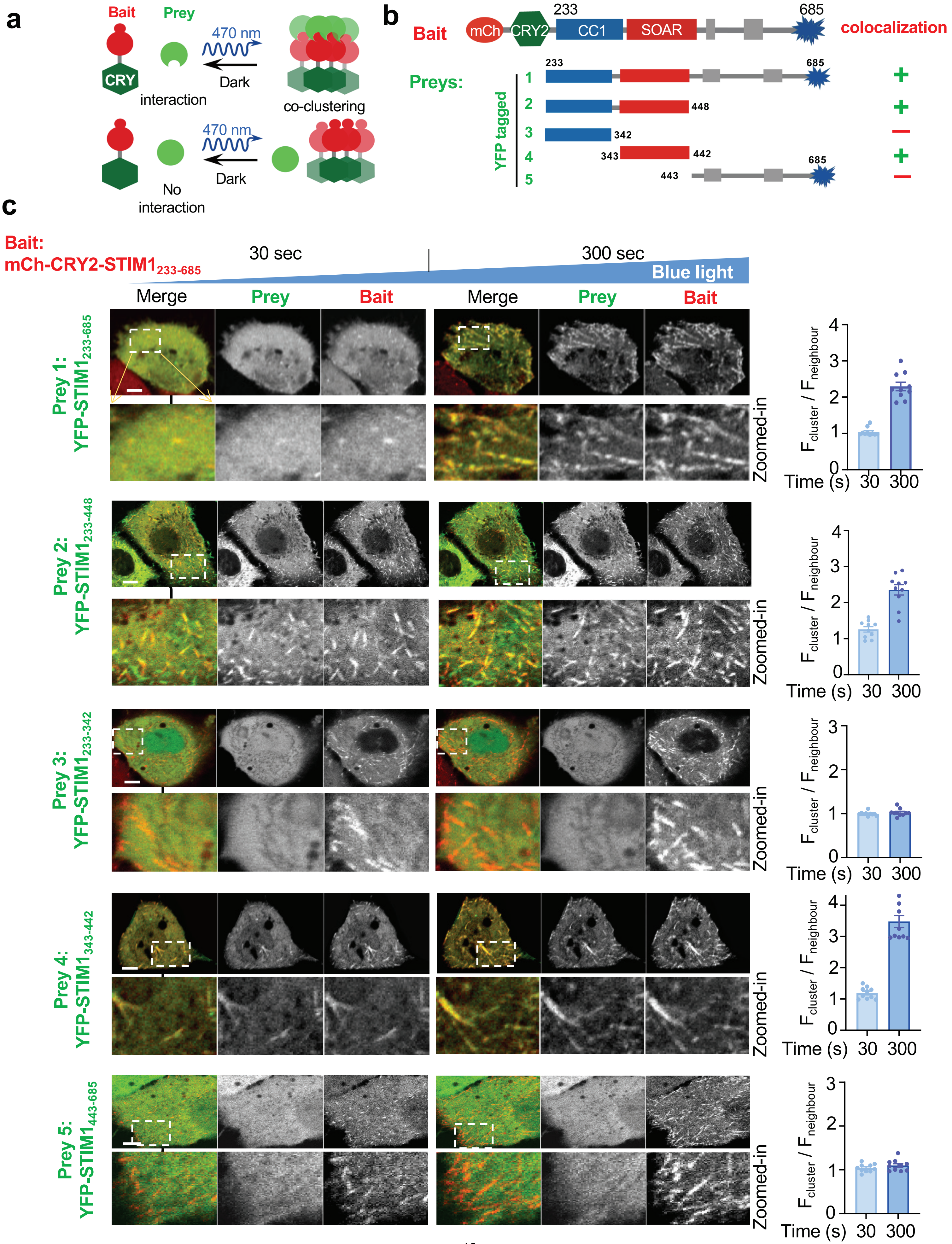

a
Bait
Prey
CRY
470 nm
Dark
interaction
co-clustering
470 nm
Dark
No interaction
b
Bait
mCh
CRY2
233
CC1
SOAR
685
colocalization
Preys:
YFP tagged
1
2
3
4
5
233
685
448
342
343
442
443
685
+
+
−
+
−
c
Bait: mCh-CRY2-STIM1233-685
30 sec
300 sec
Blue light
Merge
Prey
Bait
Prey 1: YFP-STIM1233-685
Prey 2: YFP-STIM1233-448
Prey 3: YFP-STIM1233-342
Prey 4: YFP-STIM1343-442
Prey 5: YFP-STIM1443-685
Zoomed-in
Fcluster / Fneighbour
Time (s)
30
300

Supplementary Figure 7 | Light inducible co-localization of mCh-CRY2-STIM1$_{233\text{-}685}$ (bait) with YFP-tagged STIM1ct fragments (preys). (Related to **Fig. 4**)

(**a**-**b**) Schematic of the design (**a**) and constructs used in the optogenetic co-localization assay. “+”, light-induced bait-prey co-clustering observed based on images shown in panel c; “–”, no co-clustering observed.

(**c**) Representative confocal images of HeLa cells co-expressing the bait (mCherry-CRY2-STIM1$_{233\text{-}685}$) and the indicated preys (YFP tagged STIM1ct fragments; preys 1-5) under blue light illumination (30 sec and 300 sec). Zoomed-in views of the boxed regions were also shown below the corresponding images. Quantification of the degrees of light-inducible co-clustering for the indicated preys were shown on the right. n = 10 cells. Error bars denote sem.

NOTE: Because the bait contains the S/TxIP EB1-binding motif (STIM1$_{642\text{-}645}$ targeting EB1, a microtubule plus-end tracking protein), mCh-CRY2-STIM1$_{233\text{-}685}$ exhibited comet-like distribution by tracking the plus ends of microtubules, rather than aggregation as seen with the other two baits in **Supplementary Figs. 8 and 9**, upon photoactivation. Only Preys 1, 2, and 4 containing the SOAR domain showed co-localization with the bait after blue light irradiation. Scale bar, 5 µm.

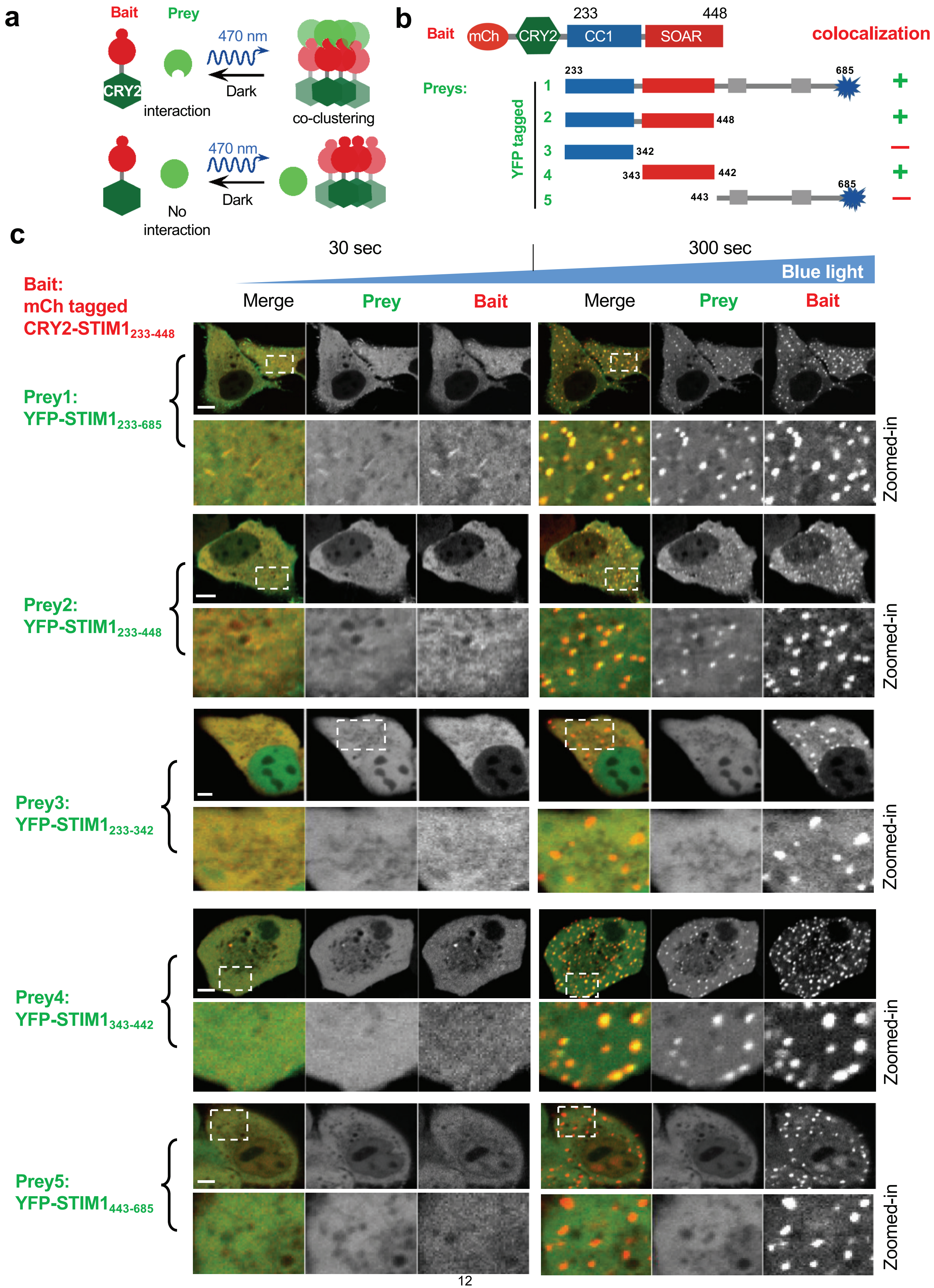

a
Bait
Prey
470 nm
Dark
CRY2
interaction
co-clustering
No interaction
b
Bait
mCh
CRY2
CC1
SOAR
233
448
colocalization
Preys:
YFP tagged
1
2
3
4
5
342
343
442
443
685
+
−
c
30 sec
300 sec
Blue light
Merge
Prey
Bait
Bait: mCh tagged CRY2-STIM1233-448
Prey1: YFP-STIM1233-685
Prey2: YFP-STIM1233-448
Prey3: YFP-STIM1233-342
Prey4: YFP-STIM1343-442
Prey5: YFP-STIM1443-685
Zoomed-in

d

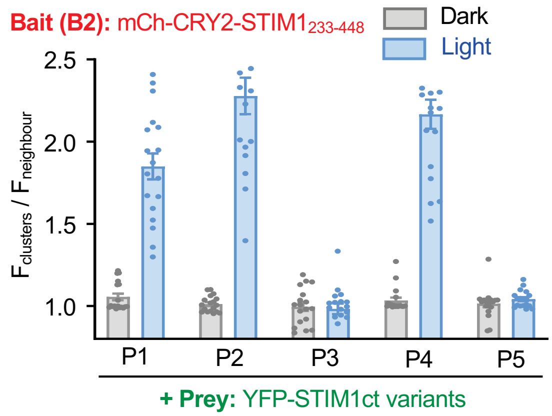

Supplementary Figure 8 | Light inducible co-clustering of mCh-CRY2-STIM1$_{233\text{-}448}$ (bait) with YFP-STIM1ct fragments (preys). (Related to **Fig. 4**)

(**a**-**b**) Schematic of CRY2 fused bait protein co-clustering with prey proteins (**a**) and the bait-prey combinations used in the assay (**b**).

(**c**) Representative confocal images of HeLa cells expressing the bait, mCherry-CRY2-STIM1$_{233\text{-}448}$, and the indicated YFP-fused preys upon blue light illumination for 30 sec (left) or 300 sec (right). The selected regions (dashed rectangle) were enlarged for better visualization of the bait-prey colocalization. YFP-STIM1$_{233\text{-}685}$ (Prey 1), YFP-STIM1$_{233\text{-}448}$ (Prey 2) and YFP-STIM1$_{343\text{-}442}$ (Prey 4) displayed light-inducible co-clustering with the bait (STIM1$_{233\text{-}448}$). Scale bar, 5 µm.

(d) Quantification of the degrees of light-inducible co-clustering for the five indicated preys in HeLa cells co-transfected with the bait. n = 18 cells from three independent experiments. (Also shown in **Figure 4e**)

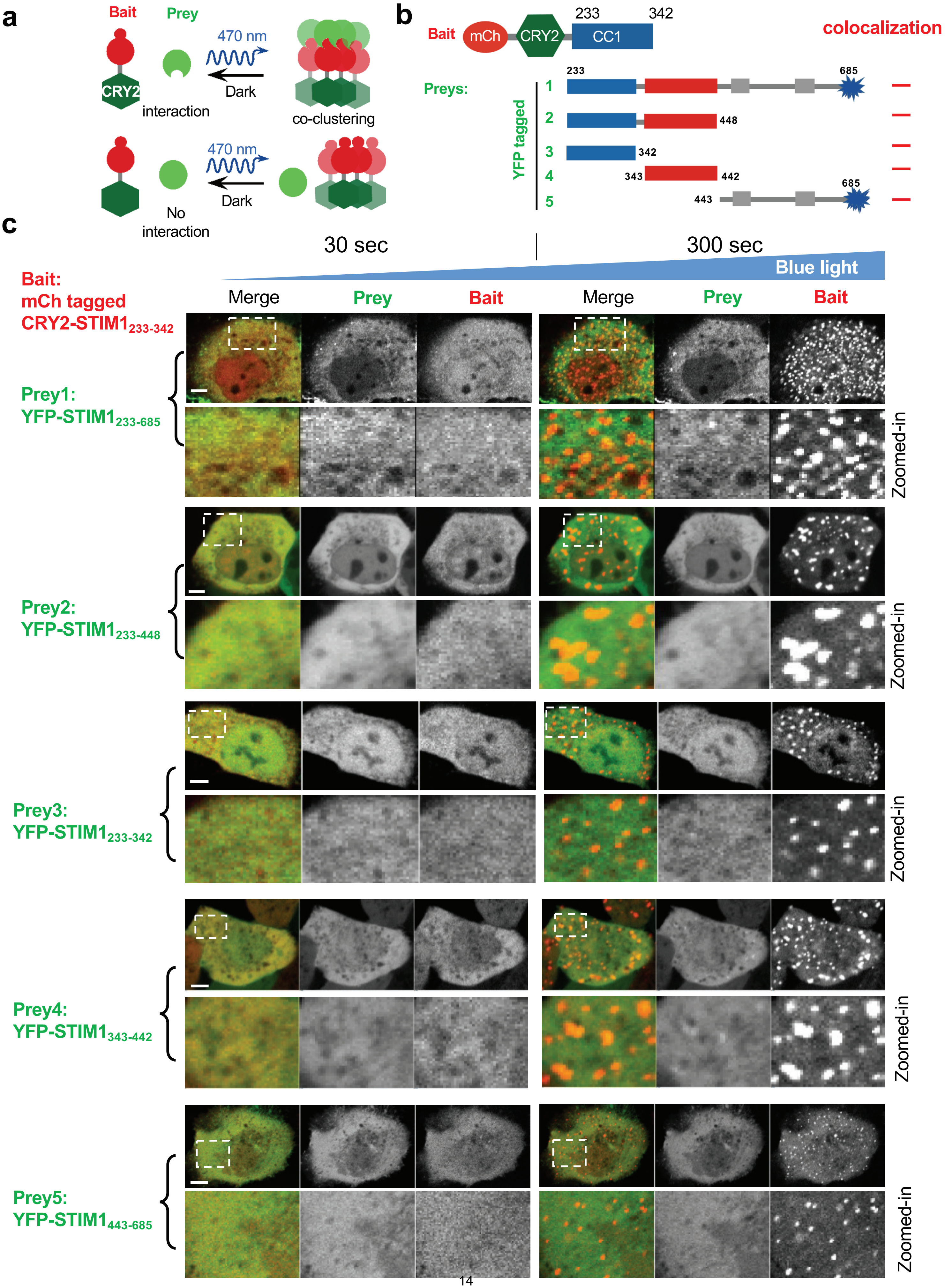

a
Bait
Prey
470 nm
Dark
CRY2
interaction
co-clustering
470 nm
Dark
No interaction
b
Bait
mCh
CRY2
CC1
233
342
colocalization
Preys:
YFP tagged
1
2
3
4
5
233
685
448
342
343
442
443
685
c
30 sec
300 sec
Blue light
Bait:
mCh tagged
CRY2-STIM1233-342
Merge
Prey
Bait
Merge
Prey
Bait
Prey1:
YFP-STIM1233-685
Prey2:
YFP-STIM1233-448
Prey3:
YFP-STIM1233-342
Prey4:
YFP-STIM1343-442
Prey5:
YFP-STIM1443-685
Zoomed-in
Zoomed-in
Zoomed-in
Zoomed-in
Zoomed-in

d

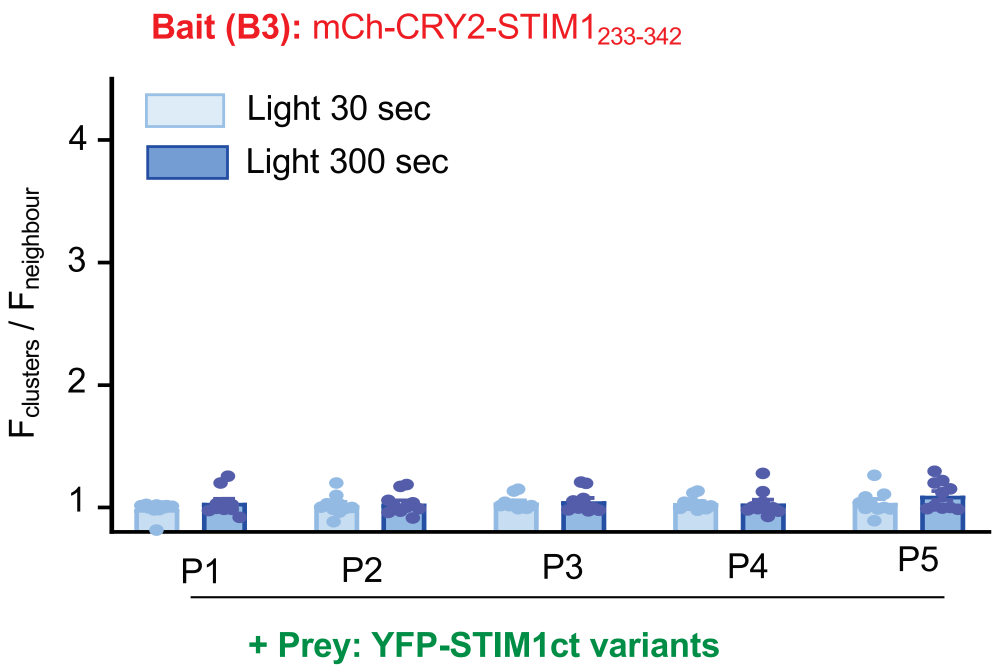

Supplementary Figure 9 | Light inducible co-clustering of mCh-CRY2-STIM1$_{233\text{-}342}$ (bait) with the indicated YFP-STIM1ct fragments (preys). (Related to **Fig. 4**) Error bars denote sem.

(**a**-**b**) Schematic of the design (**a**) and the bait-prey combinations (**b**) used in the co-clustering assay.

(**c**) Representative confocal images of HeLa cells expressing the bait, mCherry-CRY2-STIM1$_{233\text{-}342}$ and the indicated YFP-tagged preys under blue light illumination. All the preys did not show co-clustering with the bait after photo-illumination at 470 nm. Scale bar, 5 µm.

(**d**) Quantification of the degrees of light-inducible co-clustering for the indicated preys. n = 10 cells.

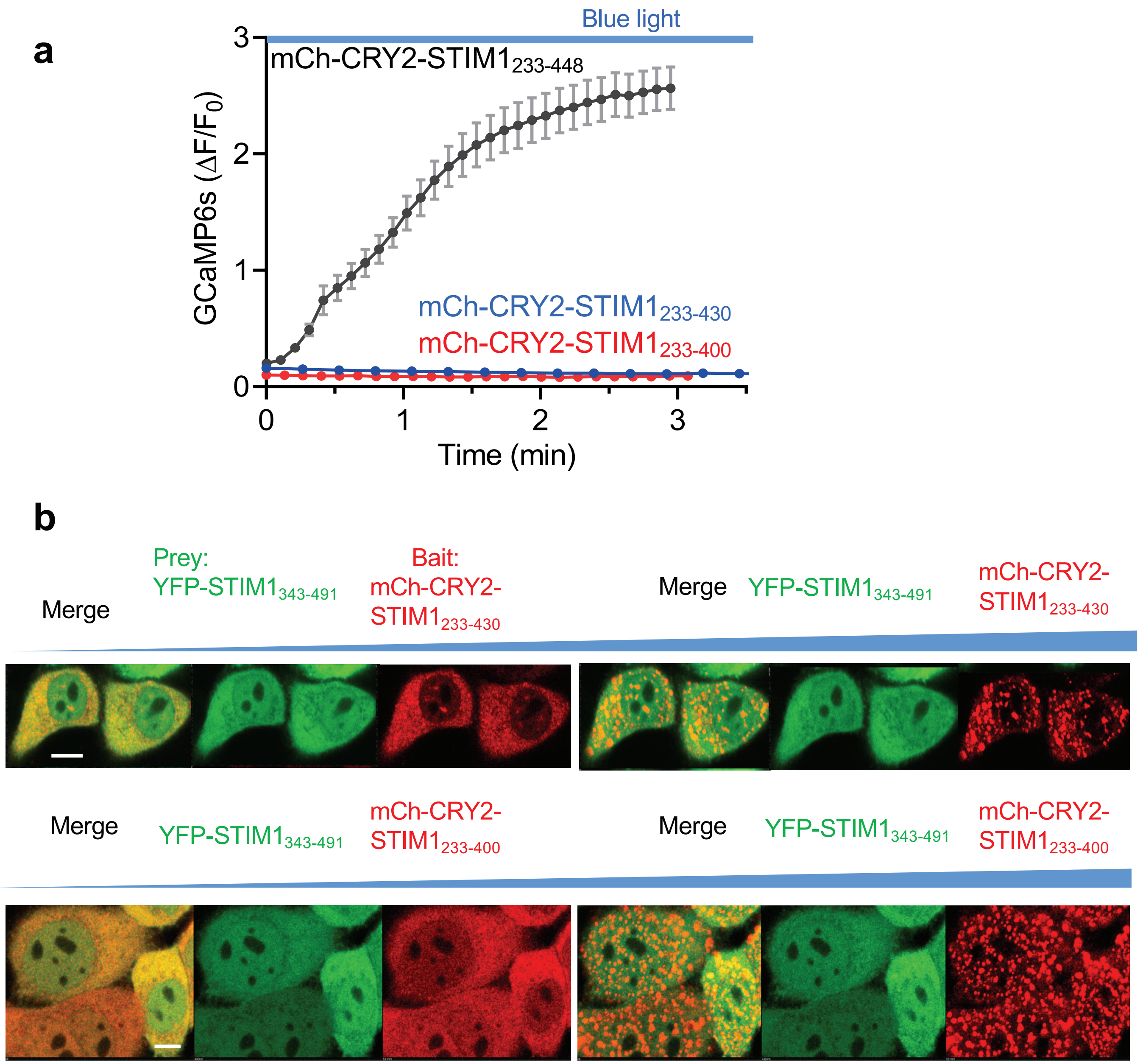

Supplementary Figure 10 | Truncation of the SOAR domain abolished Ca^{2+} influx and STIM1 oligomerization. (Related to Figs. 1-4)

(**a**) Time courses of blue light-triggered Ca^{2+} entry in HeLa-GCaMP6s cells co-transfected with mCh-CRY2-$STIM1_{233-448}$, mCh-CRY2-$STIM1_{233-430}$ or mCh-CRY2-$STIM1_{233-400}$. n= 30 cells. Error bars denote sem.

(**b**) Representative confocal images of HeLa cells expressing YFP-$STIM1_{343-491}$ (within intact SOAR) and SOAR-truncated variants, mCh-CRY2-$STIM1_{233-430}$ (*up*) or mCh-CRY2-$STIM1_{233-400}$ (*bottom*) after blue light illumination for 30 sec (left) or 300 sec (right). Scale bar 5 µm.

NOTE: We failed to detect the light-induced co-clustering between the baits and the prey, implying that the structural integrity of SOAR is required for STIM1 oligomerization.

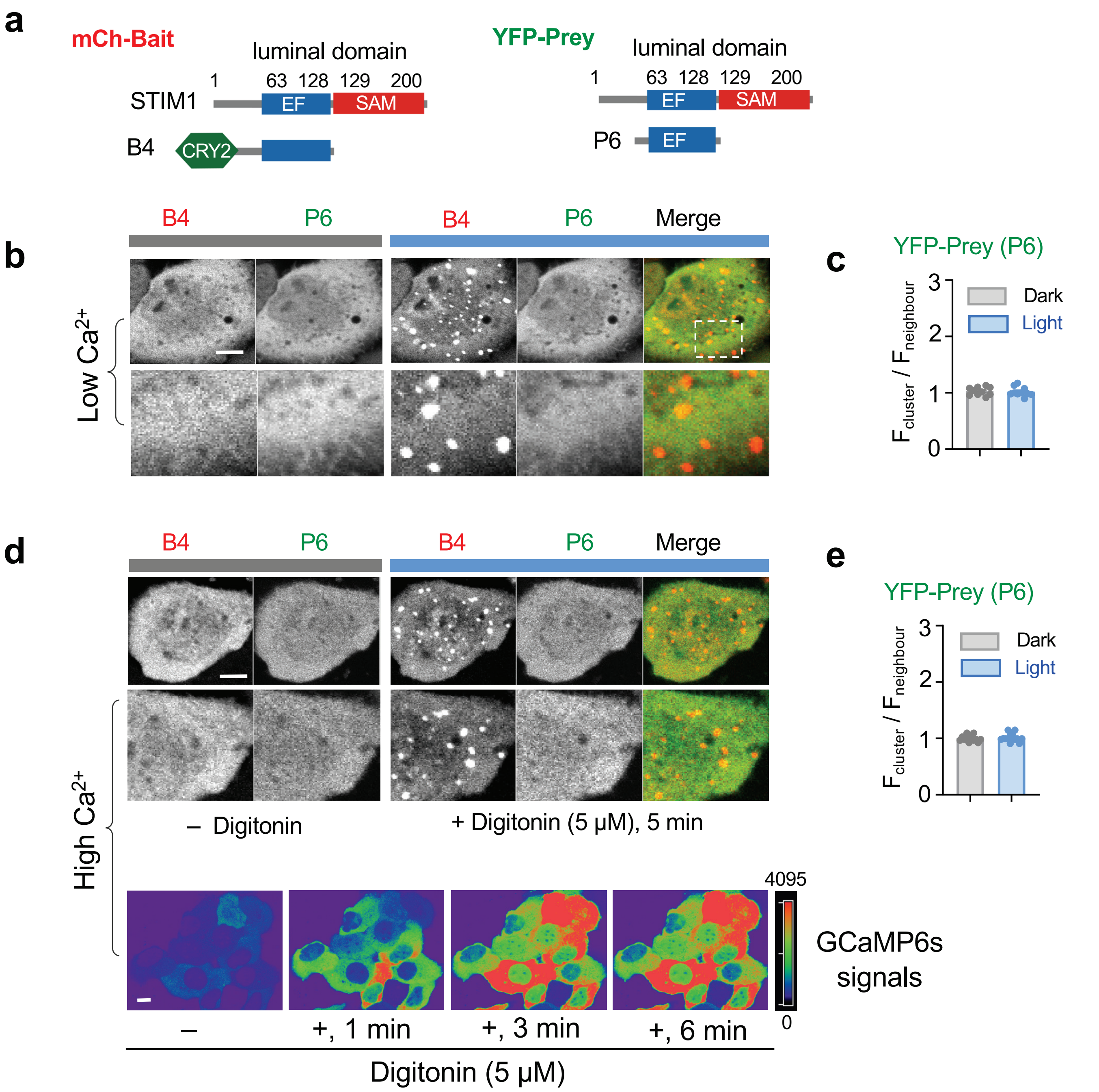

Supplementary Figure 11 | Evaluating the effect of Ca^{2+} on STIM1 EF-hand/EF-hand interactions. (Related to **Fig. 4**) Scale bar, 5 μm. Error bars denote sem.

(**a**) Schematic of the bait-prey combination.

(**b**-**c**) Representative confocal images of HeLa cells expressing the bait, mCherry-CRY2-EF (B4, $STIM1_{32\text{-}128}$) and YFP-tagged EF-hand (YFP-EF; P6) before and after blue light illumination. Note that panel b was also shown in **Fig. 4h**. YFP-EF did not show co-clustering with the bait after photo-illumination. The clustering of P6 was measured by the ratio of $F_{cluster}/F_{neighbor}$ in panel **c**. n = 10 cells.

(**d**-**e**) Representative confocal images of HeLa cells expressing B4 and P6 following treatment with 5 μM digitonin to allow extracellular Ca^{2+} influx (1.8 mM). (**d**) The bottom images showed Ca^{2+} entry after treatment of 5 μM digitonin and cells remained intact within the first 6 min. The top images showed that YFP-EF did not co-cluster with the baits after blue light illumination even in the presence of digitonin (with subsequent high Ca^{2+} loading into the cytosol). (**e**) Quantification of the clustering degrees of the prey. n = 10 cells.

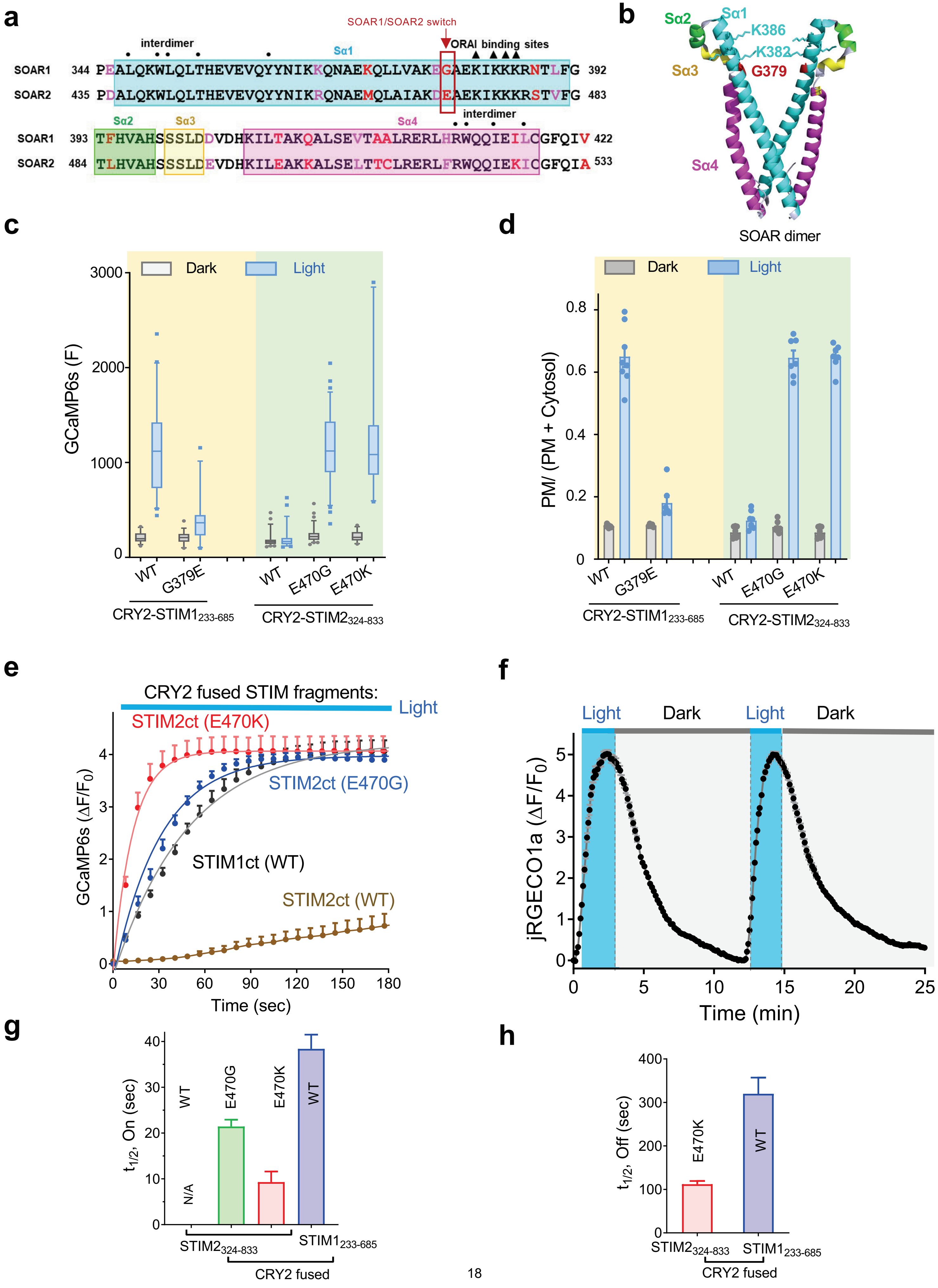
a
SOAR1/SOAR2 switch
interdimer
Sα1
ORAI binding sites
SOAR1 344 PEALQKWLQLTHEVEVQYYNIKKQNAEKQLLVAKEGAEKIKKKRNTLFG 392
SOAR2 435 PDALQKWLQLTHEVEVQYYNIKRQNAEMQLAIAKDEAEKIKKKRSTVFG 483
Sα2
Sα3
Sα4
interdimer
SOAR1 393 TFHVAHSSSLDDVDHKILTAKQALSEVTAALRERLHRWQQIEILCGFQIV 422
SOAR2 484 TLHVAHSSSLDEVDHKILEAKKALSELTTCLRERLFRWQQIEKICGFQIA 533
b
Sα2
Sα1
K386
K382
Sα3
G379
Sα4
SOAR dimer
c
Dark
Light
GCaMP6s (F)
WT
G379E
WT
E470G
E470K
CRY2-STIM1233-685
CRY2-STIM2324-833
d
Dark
Light
PM/ (PM + Cytosol)
WT
G379E
WT
E470G
E470K
CRY2-STIM1233-685
CRY2-STIM2324-833
e
CRY2 fused STIM fragments:
Light
STIM2ct (E470K)
STIM2ct (E470G)
STIM1ct (WT)
STIM2ct (WT)
GCaMP6s (ΔF/F0)
Time (sec)
f
Light
Dark
Light
Dark
jRGECO1a (ΔF/F0)
Time (min)
g
t1/2, On (sec)
WT
N/A
E470G
E470K
WT
STIM2324-833
STIM1233-685
CRY2 fused
h
t1/2, Off (sec)
E470K
WT
STIM2324-833
STIM1233-685
CRY2 fused

Supplementary Figure 12 | An optogenetic approach to dissect the molecular determinants in STIM1 and STIM2 that account for differential ORAI activation. Data were shown as mean ± sem. (Related to **Fig. 5**)

(**a**) Sequence alignments of the SOAR domains derived from STIM1 (SOAR1) and STIM2 (SOAR2).

(**b**) The 3D structure of SOAR1 highlighting the key residue (PDB entry: 3TEQ, G379 in STIM1; the equivalent of E470 in STIM2) responsible for the differential ORAI-activating capabilities of STIM proteins.

(**c**-**d**) Quantification of light induced Ca^{2+} influx (**c**) and the cytosol-to-PM translocation (**d**) of CRY2 fused STIM1ct ($STIM1_{233\text{-}685}$) or STIM2ct ($STIM2_{324\text{-}833}$). n = 60 cells for Ca^{2+} influx from three independent experiments. n = 10 cells for PM translocation.

(**e**) Time courses of blue light-triggered Ca^{2+} entry in HeLa-GCaMP6s cells co-transfected with mCh-CRY2-STIMct variants and CFP-ORA1. n = 60 cells from three independent experiments.

(**f**) Reversible Ca^{2+} response in HeLa cells co-expressing CRY2-STIM2ct (E470K) and the red Ca^{2+} sensor jRGECO1a upon exposure to repeated light-dark cycles (2 min ON + 12 min OFF). Blue bar, light stimulation at 470 nm with a power density of 4 mW/cm^2. n = 24 cells.

(**g**-**h**) Comparison of the activation (**g**) and deactivation half-lives (**h**) of CRY2-STIM1ct (aa 233-685) and CRY2-STIM2ct-E470K or E470G (aa 324-833). n = 24 cells.

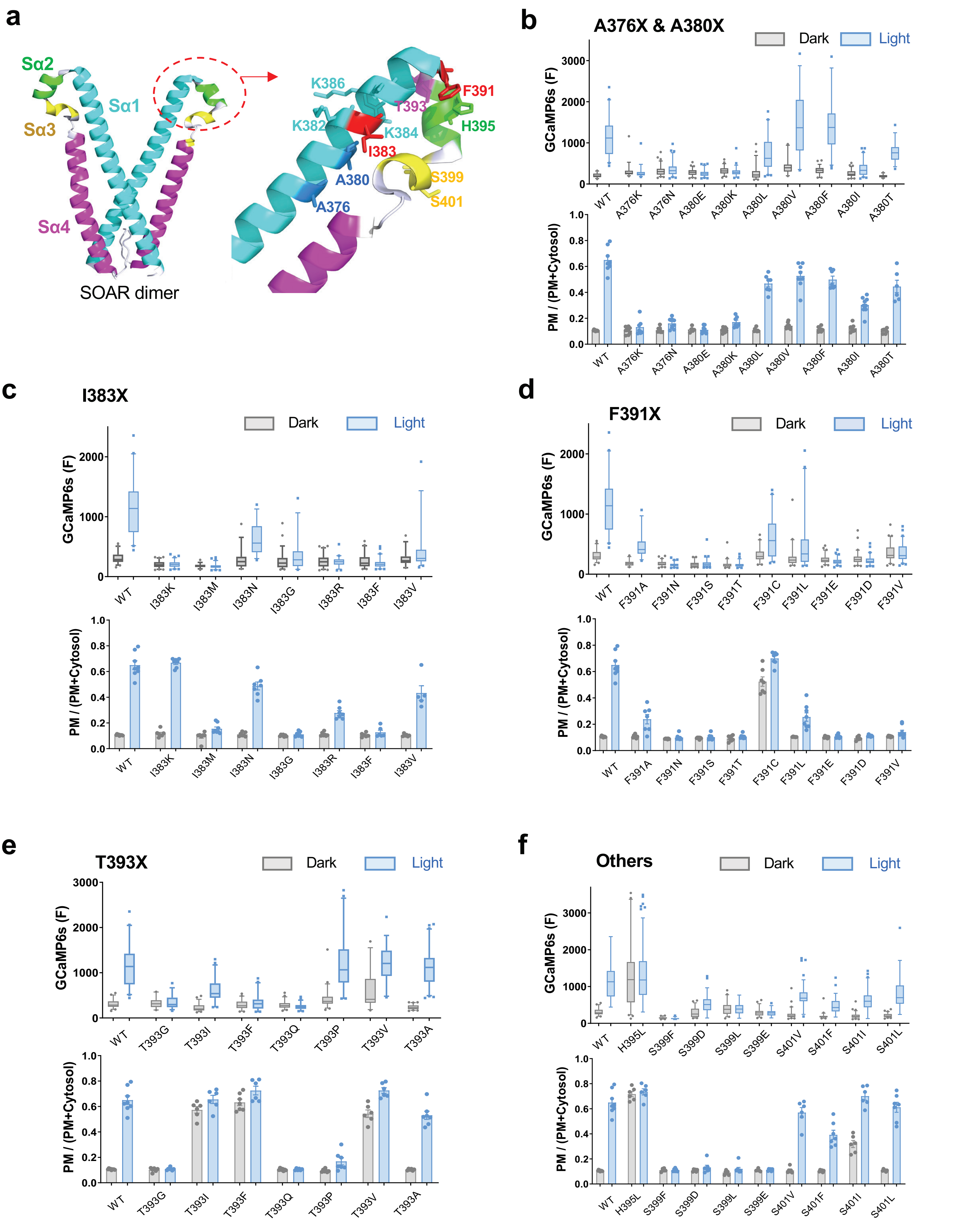

a
Sα2
Sα1
Sα3
Sα4
SOAR dimer
K386
F391
T393
K382
K384
H395
I383
A380
S399
A376
S401
b
A376X & A380X
Dark
Light
GCaMP6s (F)
PM / (PM+Cytosol)
WT
A376K
A376N
A380E
A380K
A380L
A380V
A380F
A380I
A380T
c
I383X
I383K
I383M
I383N
I383G
I383R
I383F
I383V
d
F391X
F391A
F391N
F391S
F391T
F391C
F391L
F391E
F391D
F391V
e
T393X
T393G
T393I
T393F
T393Q
T393P
T393V
T393A
f
Others
H395L
S399F
S399D
S399L
S399E
S401V
S401F
S401I
S401L

Supplementary Figure 13 | Optogenetic screening of SOAR mutations that decouple ORAI binding from ORAI channel gating. (related to **Fig. 5**)

(**a**) Mutations in STIM1 were mapped to the 3D structure of SOAR.

(**b**-**f**) Summary of the screening results. The HeLa-GCaMP6s stable cells were co-transfected with mCh-tagged STIM1ct mutants and CFP-ORAI1. Box-whisker plots showing the quantification of light induced Ca^{2+} influx (top) and cytosol-to-PM translocation (bottom), as well as photo-induced PM translocation, for CRY2 fused STIM1ct variants. Box-whisker plots are expressed as median, interquartile range with 5-95 percentile distribution. GCaMP6s was used to report the basal level of Ca^{2+} (grey) and light induced Ca^{2+} influx (blue) in HeLa cells. The I383K mutant showed light-inducible PM translocation without Ca^{2+} influx (panel c). The T393F mutant exhibited constitutive localization at PM without eliciting significant changes in Ca^{2+} influx upon photoactivation (panel e). Some data were also used in the scatter plot shown in **Fig. 5c**. n = 60 cells from three independent experiments for Ca^{2+} signals. n = 10 for cytosol-to-PM translocation. Error bars denote sem.

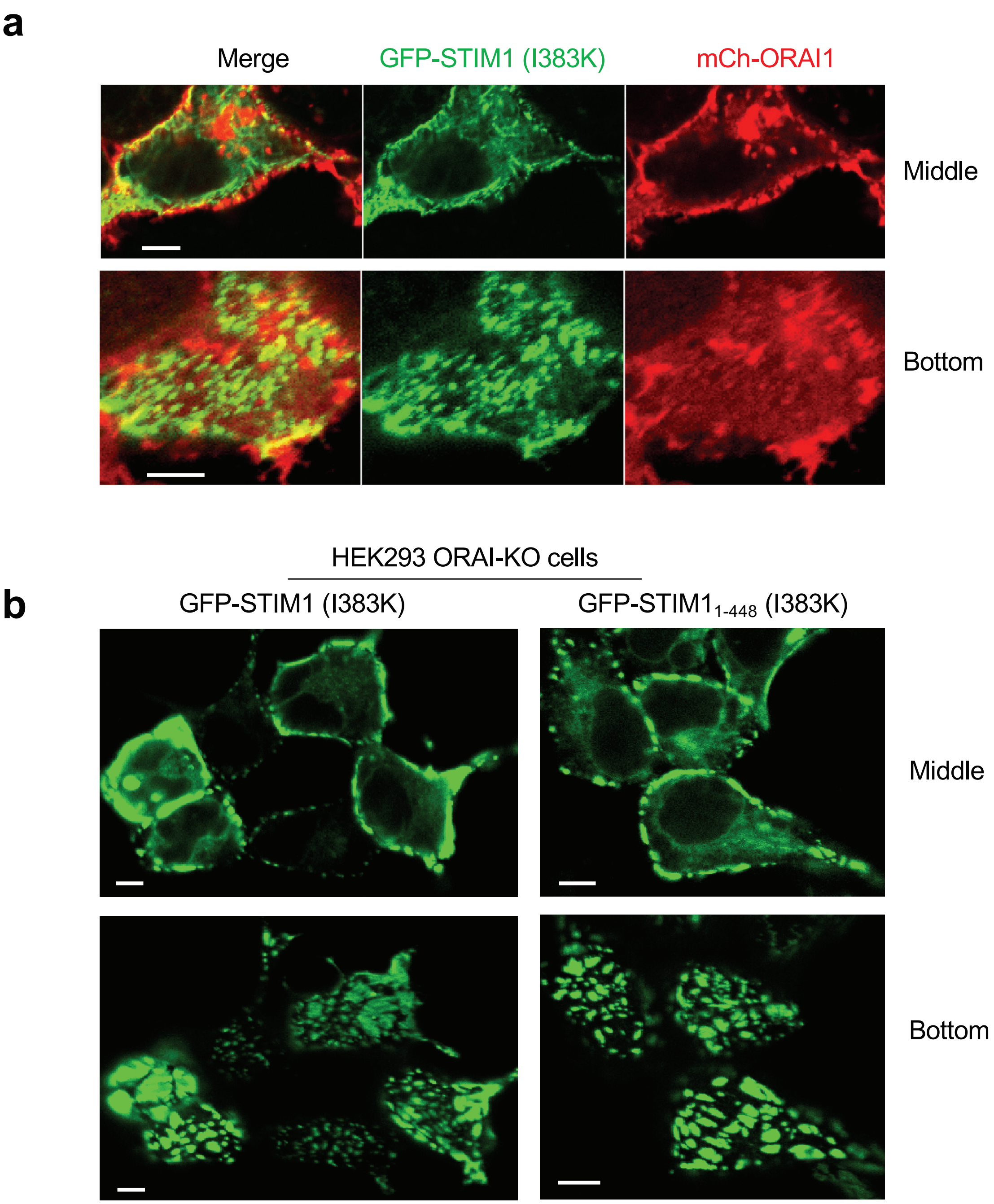

Supplementary Figure 14 | I383K caused constitutive formation of STIM1 puncta at ER-PM junctions. (related to **Fig. 5**) Scale bar 5 µm.

(**a**) Representative confocal images (top: the middle plane; bottom: the bottom layer) of HeLa cells co-expressing GFP-STIM1-I383K and mCh-ORAI1 at the resting condition. The footprint view of the image indicated very little colocalization between GFP-STIM1 and mCh-ORAI1.

(**b**) Representative middle (*top*) and footprint (*bottom*) views of confocal images of ORAI-deficient HeLa cells expressing GFP-STIM1-I383K and GFP-STIM1$_{1\text{-}448}$ -I383K, the latter of which lacks the phosphoinositide (PIP)-binding polybasic tail in the C-terminus. We speculate that the I-to-K substitution generates a new PIP-binding motif to enable the spontaneous translocation of STIM1 toward PM even in the absence of ORAI1 or STIM1-PB.

a

SOAR mutations present in patients

Mutations	Cancer genomics[1] Sample ID	Cancer genomics[1] Cancer type	Other diseases	Optogenetic dissection
W350L	TCGA-35-4123-01	Lung Adenocarcinoma		
E378F	TCGA-F1-A448-01	Stomach Adenocarcinoma Mucinous Stomach Adenocarcinoma		
E378S	TCGA-F1-A448-01	Stomach Adenocarcinoma Mucinous Stomach Adenocarcinoma		
A380T	Pat_41_Post	Melanoma		
K382N	TCGA-19-5956-01	Glioblastoma Multiforme		
K384Q	587376	Colorectal Adenocarcinoma		
H395Y	TCGA-44-2656-01	Lung Adenocarcinoma		Gain of function
V396M	TCGA-AX-A0J1-01	Uterine Endometrioid Carcinoma		
L402R	TCGA-DW-7963-01	Papillary Renal Cell Carcinoma		
K408T	587376	Colorectal Adenocarcinoma		
K408Q	TCGA-D3-A2JF-06	Cutaneous Melanoma		
R424W	TCGA-BR-4368-01	Stomach Adenocarcinoma		Gain of function
R426L	H072969	Hepatocellular Adenoma		Loss of function
R426C	MEL-IPI_Pat157-Tumor-SM-7A15B	Cutaneous Melanoma	Dental enamel maturation defect, Mild immunodeficiency[2]	Loss of function
R429C			Muscular hypotonia, partial iris hypoplasia / mydriasis, enuresis[2]	Loss of function
E434K	TCGA-AN-A046-01, TCGA-IB-7651-01,	Breast Invasive Ductal Carcinoma, Pancreatic Adenocarcinoma		

1. The cancer genomics data is from cBioPortal: http://www.cbioportal.org/results/mutations?session_id=5b9ab9a3498eb8b3d567d6c1
2. Lacruz RS, Feske S. Diseases caused by mutations in ORAI1 and STIM1. Ann N Y Acad Sci. 2015 Nov;1356:45-79

b

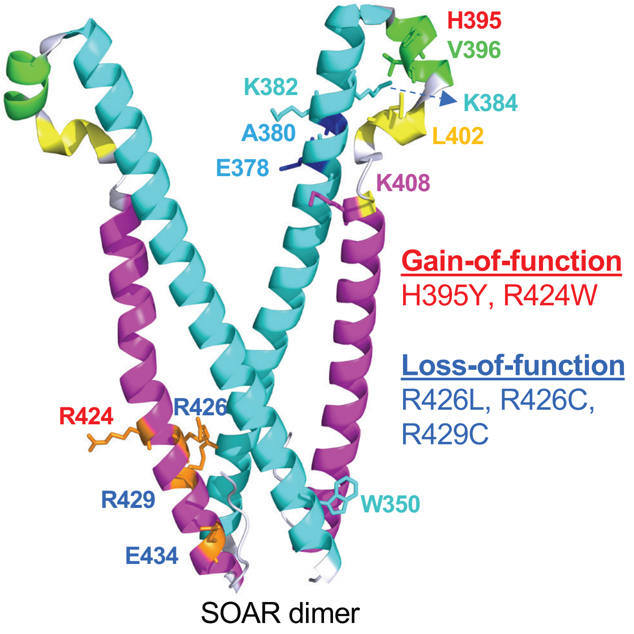

c

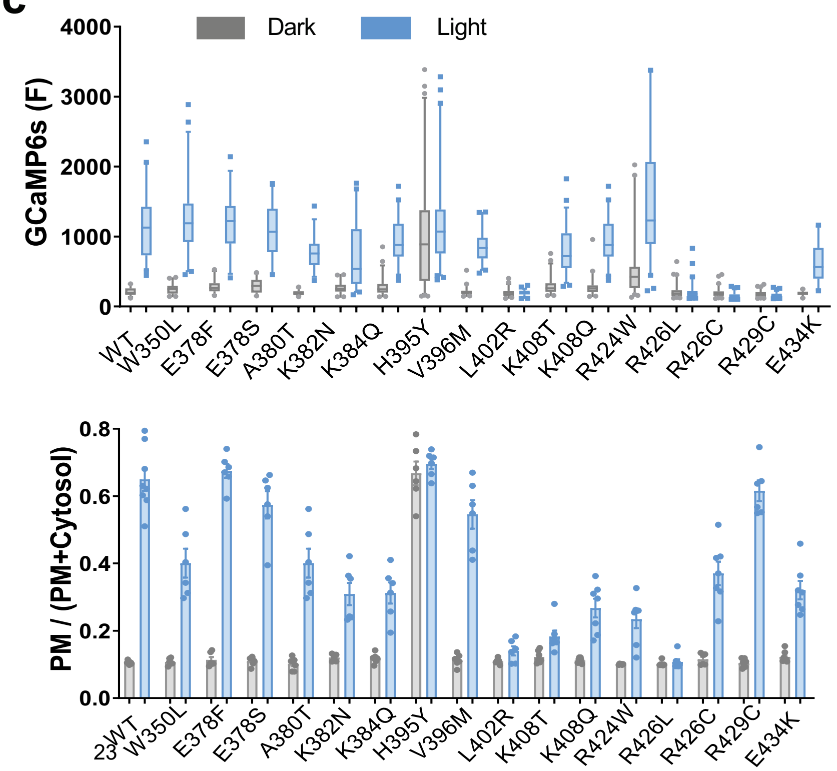

Supplementary Figure 15 | Optogenetic dissection of cancer-associated mutations in the SOAR domain. (related to **Fig. 5**)

(**a**) A summary of SOAR mutations found in cancer patients.

(**b**) Some cancer-associated mutations of STIM1 were mapped to the 3D structure of SOAR. Gain- and loss-of-function mutations validated in this study were highlighted in red and blue, respectively.

(**c**) Quantification of light induced Ca^{2+} influx (top) and cytosol-to-PM translocation (bottom) for CRY2 fused STIM1ct variants. Ca^{2+} influx were shown in Box-whisker plots, which are expressed as median, interquartile range with 5-95 percentile distribution (n = 60 cells). Cytosol-to-PM translocation were plotted as scatter plot (n = 10 cells). Error bars, sem.

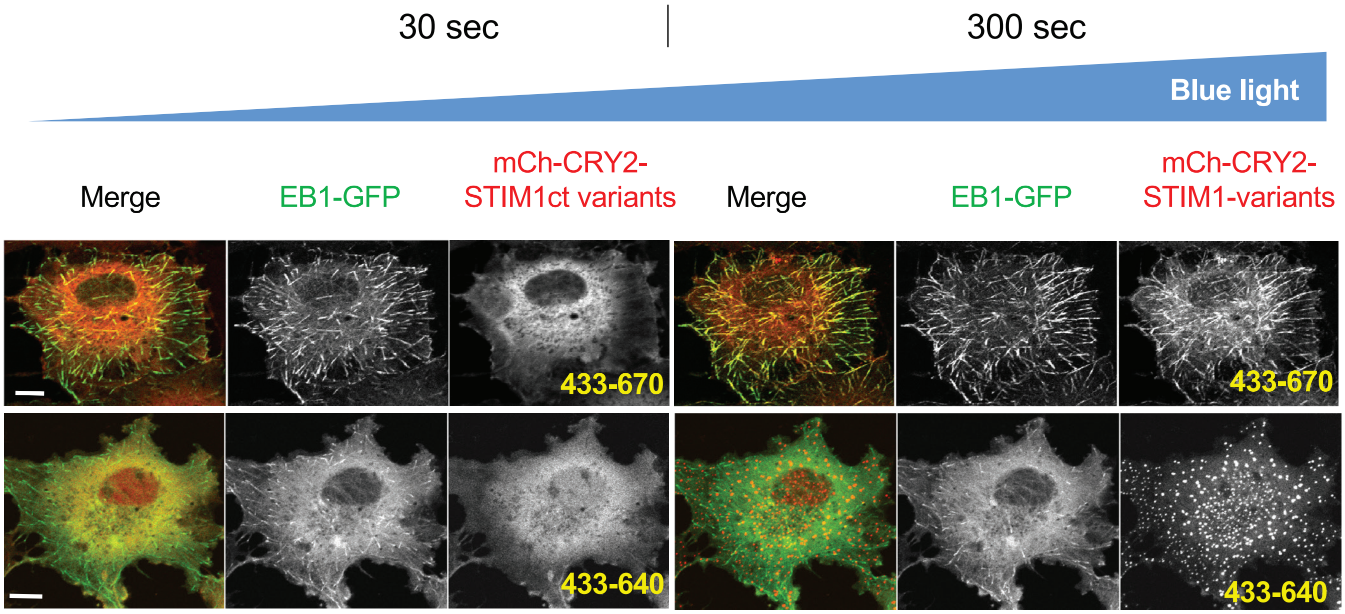

Supplementary Figure 16 | CRY2-based optogenetic tools to characterize the EB1 binding domain of STIM1. (Related to **Fig. 6**)

The representative confocal images showing light inducible +TIPs tracking in COS-7 cells co-expressing EB1-GFP (green) and mCh-CRY2-STIM1ct fragments (red). CRY2 fused $STIM1_{443\text{-}670}$ displayed strong +TIP tracking with long "comets" formation. However, CRY2 fused $STIM1_{443\text{-}640}$, lacking the S/TxIP motif, failed to track the MT tips and tended to self-aggregate. Scale bar, 5 μm.

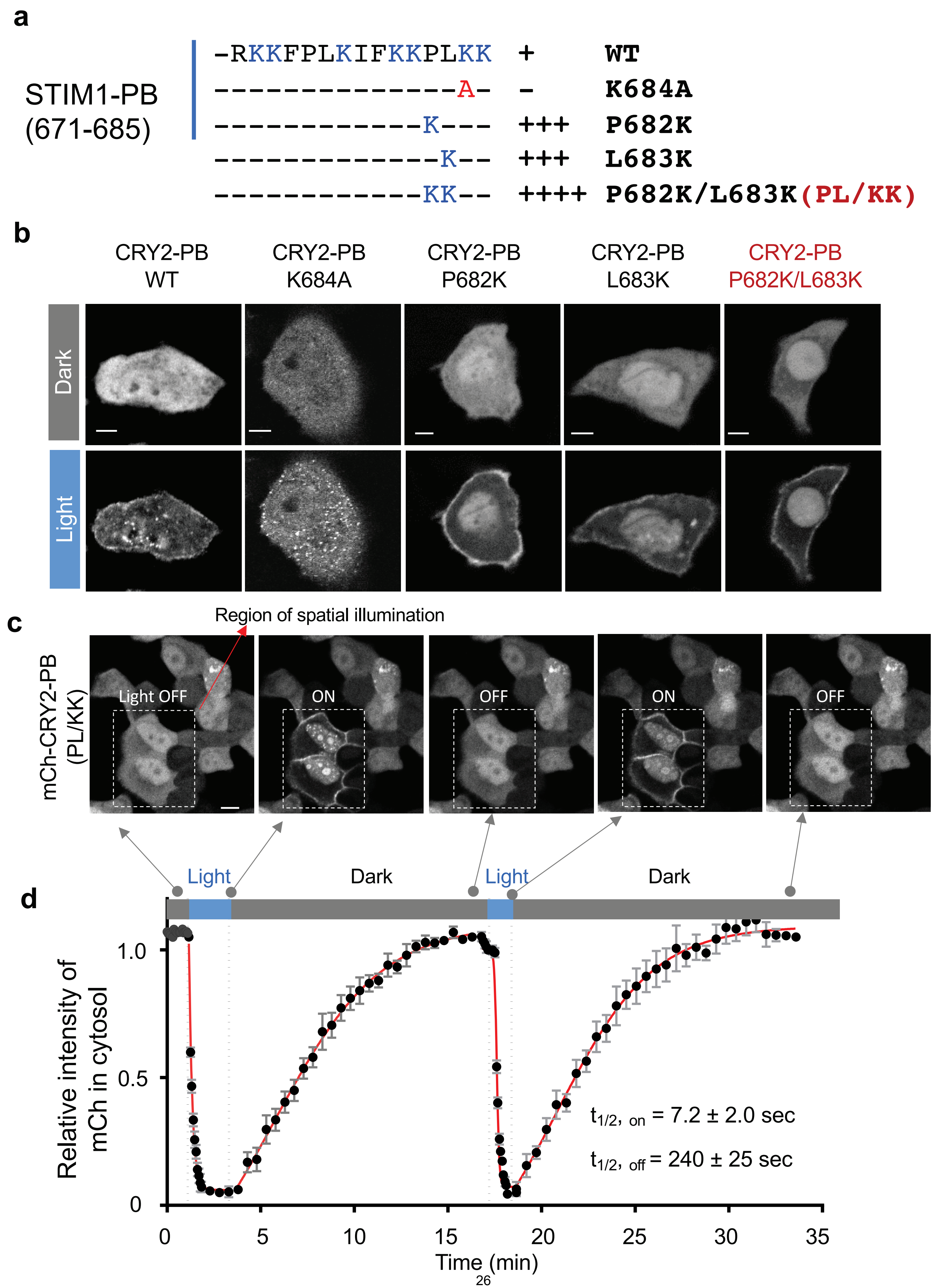
a
STIM1-PB
(671-685)
-RKKFPLKIFKKPLKK + WT
--------------A- - K684A
-------------K--- +++ P682K
--------------K-- +++ L683K
-------------KK-- ++++ P682K/L683K(PL/KK)
b
CRY2-PB WT
CRY2-PB K684A
CRY2-PB P682K
CRY2-PB L683K
CRY2-PB P682K/L683K
Dark
Light
c
Region of spatial illumination
mCh-CRY2-PB (PL/KK)
Light OFF
ON
OFF
ON
OFF
d
Light
Dark
Light
Dark
Relative intensity of mCh in cytosol
Time (min)
t1/2, on = 7.2 ± 2.0 sec
t1/2, off = 240 ± 25 sec

Supplementary Figure 17 | Optogenetic dissection of the interaction between STI1M-PB and the PM. (Related to **Fig. 6**)

(**a**) Schematic diagram showing the primary sequence of the polybasic domain (PB) of STIM1 (STIM1-PB; aa 671-685) and mutations introduced to reduce (K>A) or enhance PM-targeting (PL>KK).

(**b**) Representative confocal images of HeLa cells expressing the indicated mCh-CRY2-PB variants before (gray bar) and after blue light illumination (blue bar). Scale bar, 5 µm. NOTE: Because the PB domain resembles nuclear localization sequences that are rich in positively-charged residues, CRY2-PB variants showed both PM-like and nuclear localization.

(**c**) Spatial control of PM targeting of mCh-CYR2-PB (PL>KK). HeLa Cells were subjected to patterned blue light exposure within the selected regions. Only the illuminated region exhibited reversible cytosol-to-PM translocation, indicating the high spatial and temporal precision of the tool. Also see **Supplementary Video 5.** Scale bar, 5 µm.

(**d**) Quantification of cytosolic mCherry signals in HeLa cells expressing mCh-CRY2-PB (PL>KK) when responding to two repeated light-dark cycles. The activation and deactivation half-lives were determined to be: $t_{1/2,\ on}$ = 7.2 ± 2.0 sec and $t_{1/2,\ off}$ = 240 ± 25 sec. Blue bar, light stimulation at 488 nm. n = 7 cells. Error bars denote sem.

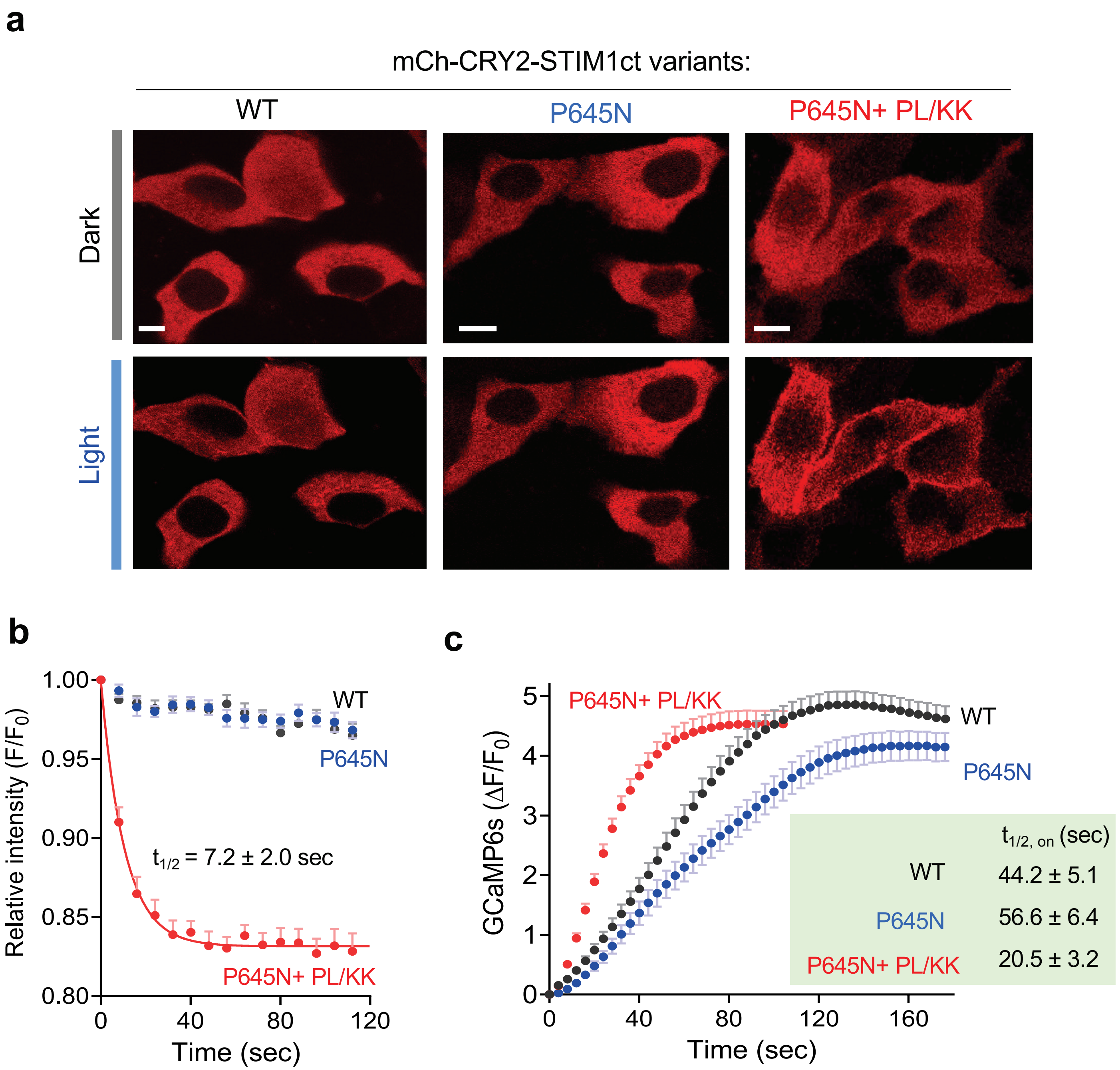

Supplementary Figure 18 | Characterization of CRY2-STIM1ct variants with mutations in the S/TxIP and PB domains. (Related to Fig. 6)

(**a**) Representative confocal images of HeLa cells expressing mCh-CRY2-STIM1ct (WT), P645N and P645N+P682K/L683K (PL/KK) before and after blue light illumination. Scale bar, 10 µm.

(**b**) The time course of light-triggered cytosol-to-PM translocation of the indicated mCh-CRY2-STIM1ct variants (WT, black; P645N, blue; P645N + PL/KK, red). Cytosolic mCherry fluorescence intensities were used to plot the curves. n = 10 cells. Error bars denote sem.

(**c**) Photo-triggered Ca^{2+} influx in HeLa cells expressing the indicated mCh-CRY2-STIM1ct variants. The half-lives of light-activated Ca^{2+} entry were listed on the right. n = 45 cells. Error bars denote sem.

a

Photoactivatable STIMct (GECAs)	ON ($t_{1/2}$, sec)	OFF ($t_{1/2}$, sec)	Related Figures
CRY2-$STIM1_{233\text{-}685}$	38.4 ± 2.1	320 ± 37.2	Supplementary Figure 12g-h
iLID-$STIM1_{233\text{-}685}$ + sspB-$STIM1_{233\text{-}685}$	28.5 ± 3.2	48.6 ± 5.4	Figure 1e
CRY2 crosslinking CIBN-$STIM1_{233\text{-}685}$	23.4 ± 2.6	153.0 ± 26.2	Figure 1h
ER-tethered CRY2-$STIM1_{233\text{-}685}$	37.8 ± 5.3	97.5 ± 16.5	Figure 1k
CRY2-$STIM2_{324\text{-}833}$	9.3 ± 2.3	112 ± 7.4	Supplementary Figure 12g-h

b

Reporter	Agonist	ER Ca^{2+} refilling ($t_{1/2}$, sec)	Reference
R-$CEPIA_{ER}$	Carbachol	~ 70	Figure 5C [4]; PMID: 29934936
D1ER	ATP, Carbachol, BHQ	~ 50	Figure 7B [5]; PMID: 22464749
D1ER	BHQ	~ 50	Figure 4A [6]; PMID: 21880734
FRET (CFP/YFP-STIM1)	BHQ	~ 30	Figure 3A [6]; PMID: 21880734
FRET (CFP/YFP-STIM1)	Histamine, BHQ	21.7 ± 2.4 s	Figure 1F [7]; PMID: 17517596

c

Reporter	Agonist	STIM1 activation ($t_{1/2}$, sec)	Reference
FRET (CFP/YFP-STIM1)	BHQ	~ 50	Figure 1A [3]; PMID: 21880734
FRET (CFP/YFP-STIM1)	Histamine, BHQ	26.5 ± 2.4 s	Figure 1C [4]; PMID: 17517596

Supplementary Figure 19 | Summary of the kinetic features of photoactivatable STIM1ct variants, ER Ca^{2+} store refilling and STIM1 activation.

(**a**) The activation (on) and deactivation (off) half-lives ($t_{1/2}$) of photoswitchable STIM1ct variants (or genetically-encoded Ca^{2+} actuators; GECAs) generated in the study.

(**b-c**) The reported half-lives ($t_{1/2}$) of ER Ca^{2+} store refilling (b) and STIM1 activation.

Supplementary Table 1. Primers used in this study.

**Fluorescent proteins fused STIM1 fragments**	
STIM1_XhoI_For	ccg ctcgag ATG GAT GTG TGC GCC CGT CTT G
STIM1_342_BamHI_Rev:	ccg GGATCCcccgaacctccATACCATGAGCTGTGTGATTC
STIM1_233_XhoI_For	cgg CTCGAG GA GGT AGT GGT GGC CAGAACCGT TACTCCAAGGAG
STIM1_343_XhoI_For	cgg CTCGAG GA GGT AGT GGT GGC GCTCCAGAG GCCCTTCAGAAG
STIM1_443_XhoI_For	cgg CTCGAG GA GGT AGT GGT GGC AACAACCCT GGCATCCACTCA
STIM1_685_EcorI_Rev	ccg gaattc CTA CTT CTT AAG AGG CTT CTT AAA AAT TTT G
STIM1_342_EcorI_Rev	Cgg GAATTC cta AGCATACCATGAGCTGTGAGATTC
STIM1_448_EcorI_Rev	Cgg GAATTC CTA GTG GAT GCC AGG GTT GTT GAC AAT
STIM1_38_XhoI_For	cgg CTCGAG GA ggtagt GCCAACTCT GAGGAGTCCACTGCA
STIM1_129_XhoI_For	cgg CTCGAG GA ggtagt GTATAC AATTGGACCGTGGATGAGGTG
STIM1_209_EcorI_Rev	cgg GAATTC CTA ATTATGGCGAGTCAAGAGAGGAGGCCCAAAGAG
STIM1_127_EcorI_Rev	cgg GAATTC CTA TGATGACTTCCATGCCTTCCACAG
**Optogenetic Modules fused STIM1 fragments**	
STIM1_BspEI-For	cgg TCCGGA ggtagt ATGGATGTATGCGTC CGTCTTGCC
STIM1_38_BspEI-For	cgg TCCGGA ggtagt GCCAACTCT GAGGAGTCCACTGCA
STIM1_128_BspEI-For	cgg TCCGGA ggtagt GTATAC AATTGGACCGTGGATGAGGTG
STIM1_200_BspEI-For	cgg TCCGGA ggtagt GGGCCTCCTCTCTTG ACTCGC
STIM1_233_BspEI-For	Cgg TCCGGA ggtagt CAGAACCGTTACTCCAAGGAG
STIM1_238_BspEI-For	Cgg TCCGGA ggtagt AAGGAGCACATGAAGAAGATGATG
STIM1_241_BspEI-For	Cgg TCCGGA ggtagt ATGAAGAAGATGATGAAGGACTTGGAG
STIM1_244_BspEI-For	Cgg TCCGGA ggtagt ATGATGAAGGACTTGGAGGGG
STIM1_248_BspEI-For	Cgg TCCGGA ggtagt TTGGAGGGGTTACACCGAGCT
STIM1_251_BspEI-For	Cgg TCCGGA ggtagt TTACACCGAGCTGAGCAGAGT
STIM1_252_BspEI-For	Cgg TCCGGA ggtagt CACCGAGCTGAGCAGAGTCTG
STIM1_255_BspEI-For	Cgg TCCGGA ggtagt GAGCAGAGTCTGCATGACCTT
STIM1_258_BspEI-For	Cgg TCCGGA ggtagt CTGCATGACCTTCAGGAAAGG
STIM1_259_BspEI-For	Cgg TCCGGA ggtagt CATGACCTTCAGGAAAGGCTG
STIM1_265_BspEI-For	Cgg TCCGGA ggtagt CTGCACAAGGCCCAGGAGGAG
STIM1_271_BspEI-For	Cgg TCCGGA ggtagt GAGCACCGCACAGTGGAGGTG
STIM1_343_BspEI-For	Cgg TCCGGA ggtagt GCTCCAGAGGCCCTTCAGAAG

STIM1_443_BspEI-For	Cgg TCCGGA ggtagt AACAACCCTGGCATCCACTCA
STIM1_671-685-For	CCGGA ggtagt CGGAAGAAGTTTCCCCTCAAAATCTTTAAGAAGCCTCTTAAGAAGT G
STIM1_671-685-For	GATCC ACTTCTTAAGAGGCTTCTTAAAGATTTTGAGGGGAAACTTCTTCCGactacc T
STIM1_685_BamHI_Rev	cgg GGATCC CTA CTT CTT AAG AGG CTT CTT AAA AAT TTT G
STIM1_670_BamHI_Rev	cgg GGATCC CTA GCCTGGGCTGGAGTCTGTTTC
STIM1_342 _BamHI_Rev	cgg GGATCC CTA AGCATACCATGAGCTGTGAGATTC
STIM1_448_BamHI_Rev	Cgg GGATCC CTA GTG GAT GCC AGG GTT GTT GAC AAT
STIM1_209_BamHI_Rev	cgg GGATCC CTA ATTATGGCGAGTCAAGAGAGGAGGCCCAAAGAG
STIM1_127 _BamHI_Rev	cgg GGATCC CTA TGATGACTTCCATGCCTTCCACAG
STIM1_336_HidIII-For	Cgg AAGCTT GAATCACACAGCTCATGGTATGCT
STIM1_486_XhoI-Rev	Cgg CTCGAG CTA GCCACCGCTTCC CGACACAATCTCCTCATCCATGTC
STIM1_685_Xhol_Rev	cgg CTCGAG CTA CTT CTT AAG AGG CTT CTT AAA AAT TTT G
BsrGI-ILID-For	Cgg TGTACA AG CCACCGGTC GCCACC CTGGCAACCACACTGGAACGG
ILID-BsrGI-Rev	Cgg TGTACA c AAAGTAATTTTCGTCGTTCGC
BsrGI-SspB-For	Cgg TGTACA AG CCACCGGTC GCCACC AGCTCCCCGAAACGCCCTAAGC
SspB-BsrGI-Rev	Cgg TGTACA c ACCAATATTCAGCTCGTCATA
BsrGI-CIBN_For	Cgg TGTACA AG CCACCGGTC ATGAATGGAGCTATA GGAGGTGAC
CIBN-BsrGI-Rev	Cgg TGTACA c ATGAATATAATCCGTTTTCTCCAA
**STIM1 mutations**	
STIM1_335_Xhol_M	AGGAAAGCAGAGAAGGAGCTCGAGTCTCACAGCTCATGGTAT
STIM1_445_Xmal_M	TTCCAGATTGTCAACAACCCCGGGATCCACTCACTGGTGG
SOAR_Random_F	cgg CTCGAG TCTCACAGCTCATGGTATGCT
SOAR_Random_R	cgg TGAGTGGATCCCGGGGTTGTT
STIM1_I383K	AAGGAGGGGGCTGAGAAGAAAAAAAAGAAGAGAAACACA
STIM1_T393F	AGAAACACACTCTTTGGCTTCTTCCACGTGGCCCACAGC
STIM1_P645N	AGCCGAAACACACGCATTAACCACCTGGCTGGCAAGAAG
STIM1_K684A	ATCTTTAAGAAGCCTCTTGCGAAG TAGGGATCCACCGGA
STIM1_P682K	CTCAAAATCTTTAAGAAGAAG CTT AAGAAG TAGGGATCC
STIM1_L683K	AAAATCTTTAAGAAGCCTAAA AAGAAGTAGGGATCCACC
STIM1_PL/KK	AAAATCTTTAAGAAGAAGAAA AAGAAGTAGGGATCCACC

Supplementary References

1. Kolossov, V.L. et al. Forster resonance energy transfer-based sensor targeting endoplasmic reticulum reveals highly oxidative environment. *Exp Biol Med (Maywood)* **237**, 652-662 (2012).
2. Morgan, B. *et al.* Multiple glutathione disulfide removal pathways mediate cytosolic redox homeostasis. *Nat Chem Biol* **9**, 119-125 (2013).
3. Gutscher, M. *et al.* Real-time imaging of the intracellular glutathione redox potential. *Nat Methods* **5**, 553-559 (2008).
4. Zheng, S.S. *et al.* Calcium store refilling and STIM activation in STIM- and Orai-deficient cell lines. *Pflug Arch Eur J Phy* **470**, 1555-1567 (2018).
5. Palty, R., Raveh, A., Kaminsky, I., Meller, R. & Reuveny, E. SARAF Inactivates the Store Operated Calcium Entry Machinery to Prevent Excess Calcium Refilling. *Cell* **149**, 425-438 (2012).
6. Shen, W.W., Frieden, M. & Demaurex, N. Local Cytosolic Ca2+ Elevations Are Required for Stromal Interaction Molecule 1 (STIM1) De-oligomerization and Termination of Store-operated Ca2+ Entry. *J Biol Chem* **286**, 36448-36459 (2011).
7. Liou, J., Fivaz, M., Inoue, T. & Meyer, T. Live-cell imaging reveals sequential oligomerization and local plasma membrane targeting of stromal interaction molecule 1 after Ca2+ store depletion. *P Natl Acad Sci USA* **104**, 9301-9306 (2007).
