# Supplementary material for: Optogenetic engineering to probe the molecular choreography of STIM1-mediated cell signaling
Source: Nat Commun. 2020 Feb 25;11:1039. doi: 10.1038/s41467-020-14841-9 (PMC7042325; doi:10.1038/s41467-020-14841-9)
Supplement: Supplementary file 3 — Description of Additional Supplementary Files [file 41467_2020_14841_MOESM3_ESM.pdf]

Description of Additional Supplementary Files

File Name: Supplementary Movie 1
Description: **CRY2 as an optical crosslinker to induce CIBN-STIM1ct activation and Ca^{2+} influx** (Related to **Fig. 1f-h**).
Dark-light cycles were applied to HeLa cells transfected with the red Ca^{2+} indicator, R-GECO1.2, YFP-CRY2 and YFP-CIBN-STIM1ct ($STIM1_{233\text{-}685}$). Shown were fluorescence signals of R-GECO1.2. Cells were exposed to blue light stimulation at 470 nm (4 mW/cm^2) or kept in the dark after light exposure.

File Name: Supplementary Movie 2
Description: **An optogenetic co-clustering assay to dissect the designed bait-prey interaction** (Related to **Fig. 4c-f**).
HeLa cells co-expressing the mCherry tagged baits (mCh-CRY2-STIM1ct variants) and YFP tagged preys (YFP-STIM1ct variants) were exposed to blue light (470 nm; 4 mW/cm^2).
Top panel: cells co-expressing B2, CRY2-$STIM1_{233\text{-}448,\ CC1\text{-}SOAR}$ and P2, YFP- $STIM1_{233\text{-}448,\ CC1\text{-}SOAR}$;
Middle panel: cells co-expressing CRY2-$STIM1_{343\text{-}448,\ SOAR}$ and P4, YFP- $STIM1_{343\text{-}448,\ SOAR}$;
Bottom panel: cells co-expressing B3, CRY2-$STIM1_{233\text{-}342,\ CC1}$ and P3, YFP- $STIM1_{233\text{-}342,\ CC1}$.

File Name: Supplementary Movie 3
Description: **Comparison of light induced Ca^{2+} influx for mCh-CRY2-STIM1ct variants (WT vs T393F).**
HeLa-GCaMP6s cells expressing mCh-CRY2-STIM1ct (WT, *left* panel) or mCh-CRY2-STIM1ct (T393F, *right* panel). The *upper* panel showed the merged confocal images of GCaMP6s and mCh-CRY2-STIM1c responding to blue light; The bottom panel showed quantitative analysis of Ca^{2+} signals in selected cells (n = 8) after photostimulation. The cells were exposed to the 488-nm excitation laser for photoactivation**.** Scale bar, 10 µm.

File Name: Supplementary Movie 4
Description: **Light-triggered reversible EB1 binding and cytosol-to-PM translocation of mCh-CRY2-$STIM1_{443\text{-}685}$.**
Dark-light cycles were applied to cells transfected with mCh-CRY2-$STIM1_{443\text{-}685}$. The blue light stimulation was set at 470 nm with a power density of 4 mW/cm^2.

File Name: Supplementary Movie 5
Description: **Spatiotemporal control of the cytosol-to-PM translocation of mCh-CRY2-PB ($STIM1_{671\text{-}685}$, PL/KK).**
Repeated light stimulation was sequentially applied to two selected regions in HeLa cells transfected with mCh-CRY2-$STIM1_{671\text{-}685}$. Photostimulation at 488 nm was used to activate mCh-CRY2-PB by taking advantage of the FRAP module (with 5% input in the GFP channel) of a Nikon A1R laser scanning confocal microscope.
