# Supplementary material for: Optogenetic engineering to probe the molecular choreography of STIM1-mediated cell signaling
Source: Nat Commun. 2020 Feb 25;11:1039. doi: 10.1038/s41467-020-14841-9 (PMC7042325; doi:10.1038/s41467-020-14841-9)
Supplement: Supplementary file 9 — Reporting Summary [file 41467_2020_14841_MOESM9_ESM.pdf]

natureresearch

Corresponding author(s): Yubin Zhou

Last updated by author(s): Jan 28, 2020

Reporting Summary

Nature Research wishes to improve the reproducibility of the work that we publish. This form provides structure for consistency and transparency in reporting. For further information on Nature Research policies, see Authors & Referees and the Editorial Policy Checklist.

Statistics

For all statistical analyses, confirm that the following items are present in the figure legend, table legend, main text, or Methods section.

n/a	Confirmed	
☐	☒	The exact sample size (*n*) for each experimental group/condition, given as a discrete number and unit of measurement
☐	☒	A statement on whether measurements were taken from distinct samples or whether the same sample was measured repeatedly
☒	☐	The statistical test(s) used AND whether they are one- or two-sided<br>*Only common tests should be described solely by name; describe more complex techniques in the Methods section.*
☒	☐	A description of all covariates tested
☒	☐	A description of any assumptions or corrections, such as tests of normality and adjustment for multiple comparisons
☐	☒	A full description of the statistical parameters including central tendency (e.g. means) or other basic estimates (e.g. regression coefficient) AND variation (e.g. standard deviation) or associated estimates of uncertainty (e.g. confidence intervals)
☒	☐	For null hypothesis testing, the test statistic (e.g. *F*, *t*, *r*) with confidence intervals, effect sizes, degrees of freedom and *P* value noted<br>*Give P values as exact values whenever suitable.*
☒	☐	For Bayesian analysis, information on the choice of priors and Markov chain Monte Carlo settings
☒	☐	For hierarchical and complex designs, identification of the appropriate level for tests and full reporting of outcomes
☒	☐	Estimates of effect sizes (e.g. Cohen's *d*, Pearson's *r*), indicating how they were calculated

Our web collection on statistics for biologists contains articles on many of the points above.

Software and code

Policy information about availability of computer code

Data collection	The microscope images were captured by using the NIS-Elements AR 4.0 (Nikon).
Data analysis	The microscope images were analyzed by using the NIS-Elements AR 4.0 (Nikon). GraphPad Prism 8 was used to analyze and plot the data. Data was analyzed in Graphpad Prism 8 and Microsoft Excel 2013.

For manuscripts utilizing custom algorithms or software that are central to the research but not yet described in published literature, software must be made available to editors/reviewers. We strongly encourage code deposition in a community repository (e.g. GitHub). See the Nature Research guidelines for submitting code & software for further information.

Data

Policy information about availability of data

All manuscripts must include a data availability statement. This statement should provide the following information, where applicable:

- Accession codes, unique identifiers, or web links for publicly available datasets
- A list of figures that have associated raw data
- A description of any restrictions on data availability

Supplementary Data are available online. The source data underlying Figures 1e, 1h, 1k, 2e, 2h, 3d-f, 4d-e, 5c-d, 5f, 5g, 5i-j and 6d, 6f, 6h and Supplementary Figures 1d, 2b, 3b, 3d, 5c-e, 5g, 6d, 7c, 9d, 10a, 11c, 11e, 12c-e, 12g-h, 13b-f, 15c, 17d, and 18b-c were provided as a Source Data file. The plasmids and all other data will be made available from the corresponding authors upon reasonable requests.

Field-specific reporting

Please select the one below that is the best fit for your research. If you are not sure, read the appropriate sections before making your selection.

☒ Life sciences ☐ Behavioural & social sciences ☐ Ecological, evolutionary & environmental sciences

For a reference copy of the document with all sections, see nature.com/documents/nr-reporting-summary-flat.pdf

Life sciences study design

All studies must disclose on these points even when the disclosure is negative.

Sample size	Sample sizes were not predetermined. At least 3 biological replicates were performed for most cases, unless otherwise noted in the figure legends.
Data exclusions	No data were excluded from the analysis
Replication	Each experiment was independently repeated at least three times with similar results.
Randomization	The detected cells or imaging views were randomly selected.
Blinding	The investigators were blinded to group allocation during data collection and analysis.

Reporting for specific materials, systems and methods

We require information from authors about some types of materials, experimental systems and methods used in many studies. Here, indicate whether each material, system or method listed is relevant to your study. If you are not sure if a list item applies to your research, read the appropriate section before selecting a response.

Materials & experimental systems

n/a	Involved in the study	
☒	☐	Antibodies
☐	☒	Eukaryotic cell lines
☒	☐	Palaeontology
☒	☐	Animals and other organisms
☒	☐	Human research participants
☒	☐	Clinical data

Methods

n/a	Involved in the study	
☒	☐	ChIP-seq
☒	☐	Flow cytometry
☒	☐	MRI-based neuroimaging

Eukaryotic cell lines

Policy information about cell lines

Cell line source(s)	HeLa, HEK293 and COS-7 cells are all obtained from ATCC
Authentication	Authenticated by the vendor with STR profiling
Mycoplasma contamination	The growing cell lines are tested for mycoplasma on a bi-weekly basis by using a PCR-based method (LookOut Mycoplasma PCR Detection Kit from Sigma). All cell lines used were contamination free.
Commonly misidentified lines (See ICLAC register)	No commonly misidentified cell lines were used
